# Supplementary material for: Nonlinear dynamic wave properties of travelling wave solutions in in (3+1)-dimensional mKdV−ZK model
Source: PLoS One. 2025 Jan 8;20(1):e0306734. doi: 10.1371/journal.pone.0306734 (PMC11709309; doi:10.1371/journal.pone.0306734)
Supplement: Attachment — Submitted filename: Review Comments PONE-D-24-25524.pdf [file pone.0306734.s001.pdf]

Review Comments

Manuscript Number: PONE-D-24-25524

I have gone through the whole manuscript titled on **“Nonlinear dynamic wave properties of travelling wave solutions in ion-acoustic waves”.**

Overall, the presentation of the article looks very good and but English grammar is not good. The manuscript is potentially publishable, but it requires major revisions, please see the points below to be addressed:

1. There are many grammatical errors. Kindly check them. (Full stop and commas)
2. What is the difference between solution sets 1 and 2 on page 12. They only differ in signs.
3. The references are not in a uniform style.
4. What are the applications of the obtained solutions?
5. What are the advantages and disadvantages of the proposed methods?
6. The introduction section is lacking drastically with the introduction to the Solitons. The authors are requested to read the following latest articles to enhance the introduction section and cite them properly.

doi.org/10.1155/2014/601961
doi.org/10.3390/sym15020360
doi: org/10.1016/j.asej.2022.101883
doi.org/10.3390/sym14122574
doi.org/10.3390/math10183400

Dr. Muhammad Shakeel
Associate Professor
Department of Mathematics, Faculty of Basic Sciences,
University of Wah, Wah Cantt. Pakistan
Email: muhammad.shakeel@uow.edu.pk
