# Supplementary material for: Nonlinear dynamic wave properties of travelling wave solutions in in (3+1)-dimensional mKdV−ZK model
Source: PLoS One. 2025 Jan 8;20(1):e0306734. doi: 10.1371/journal.pone.0306734 (PMC11709309; doi:10.1371/journal.pone.0306734)
Supplement: Attachment — Submitted filename: Report_Plos_One.pdf [file pone.0306734.s002.pdf]

A report on the paper entitled "**Nonlinear dynamic wave properties of travelling wave solutions in ion-acoustic waves**"

PLOS ONE
Manuscript Number: PONE-D-24-25524

This paper discussed the traveling wave solutions of the (3 + 1)-dimensional mKdV-ZK model using the new auxiliary equation method and the unified technique. Some traveling wave solutions in the form of rational, trigonometric, hyperbolic, and exponential functions were obtained. Moreover, some two- and three-dimensional figures were presented for some solutions. However, the authors should consider the following comments.

- The title of the paper is more general. I think that it should be changed.
- In the Abstract, please correct the grammar as I wrote in the attached file.
- Many sentences are not consistent with each other and there is no coherence between most sentences. Please check the whole manuscript for this point.
- The whole manuscript should be checked for typos and grammatical errors. An overall review is needed for fixing the grammatical and typos errors in the manuscript. Please see the attached file.
- The obtained results should be double checked.
- You need to describe the values of m, n and l in Eq. (2.4). Are they real, complex,etc?
- The determination of the value of S (in Eq. (2.4)) was described in page 10. Please put it in page 9.
- Punctuation marks should be checked throughout the paper, especially after the equations and at the end of the statements. Please see the attached file.
- The introduction should be rearranged in a manner to list the various effects considered in the study and their connection to the existing literature. These are some recent studies to include in the introduction covering various effects used in the present manuscript. I suggest some papers such as:
  https://doi.org/10.3390/fractalfract7030252
  https://doi.org/10.1142/S0218348X23401606
  https://doi.org/10.1080/00036811.2022.2027381
  https://doi.org/10.32604/cmes.2023.027344
  https://doi.org/10.1016/j.jksus.2022.102087
  https://scik.org/index.php/cmbn/article/view/8478
  https://scik.org/index.php/cmbn/article/view/8477
  https://doi.org/10.28924/2291-8639-22-2024-51
  https://doi.org/10.28924/2291-8639-21-2023-100
  https://doi.org/10.3390/fractalfract7050344
- In the comparison's section, you compared the considered model with a space-time fractional model. Is this selection correct? You need to compare your results with other results obtained on other papers for the same model.

In conclusion, I think a major correction is needed for this paper to be published in the PLOS ONE. I will consider the paper if the above comments are taken into consideration.

PLOS ONE

Nonlinear dynamic wave properties of travelling wave solutions in ion-acoustic waves

--Manuscript Draft--

**Manuscript Number:**	PONE-D-24-25524
**Article Type:**	Research Article
**Full Title:**	Nonlinear dynamic wave properties of travelling wave solutions in ion-acoustic waves
**Short Title:**	Nonlinear dynamic wave properties of travelling wave solutions in ion-acoustic waves
**Corresponding Author:**	S M Yiasir Arafat<br>Bangladesh University of Engineering and Technology<br>Dhaka, BANGLADESH
**Keywords:**	3+1-dimensional mKdV-ZK model; the new auxiliary equation method; the unified method; TWSs; soliton construction
**Abstract:**	The [[EQUATION]]-dimensional [[EQUATION]] model is an important framework for studying the dynamic behavior of waves in mathematical physics. The goal of this study is to look into more generic travelling wave solutions (TWSs) for the generalized ion-acoustic scenario in three dimensions. Rational, trigonometric, hyperbolic, and exponential solutions are concurrently generated by the new auxiliary equation and the unified techniques applied in the [[EQUATION]]-dimensional [[EQUATION]] model. We created numerous soliton solutions, including kink-shaped soliton solutions, anti-kink-shaped solutions, bell-shaped soliton solutions, periodic solutions, and solitary soliton solutions, for various values of the free parameters in the produced solutions. The attained solutions are displayed geometrically in the surface plot (3-D), contour, and combined two-dimensional (2-D) figures. The combined 2-D figure would make it easier to understand the impact of a wave of speed. These graphical representations are based on specific parameter settings, which help to grasp the model's intricate general behavior. However, the results of this research are compared with the outcomes attainable in published literature executed by other scholars. The results indicate the approach's effectiveness and reliability, making it suitable for widespread use in a range of sophisticated nonlinear models. These techniques successfully generate inventive soliton solutions for various nonlinear models, which are crucial in mathematical physics.
**Order of Authors:**	S M Yiasir Arafat
	M Asif
	M M Rahman
**Additional Information:**	
**Question**	**Response**
**Financial Disclosure**<br><br>Enter a financial disclosure statement that describes the sources of funding for the work included in this submission. Review the submission guidelines for detailed requirements. View published research articles from *PLOS ONE* for specific examples.<br><br>This statement is required for submission and **will appear in the published article** if the submission is accepted. Please make sure it is accurate.	The author(s) received no specific funding for this work.

Powered by Editorial Manager® and ProduXion Manager® from Aries Systems Corporation

**Funded studies**<br>Enter a statement with the following details:<br>• Initials of the authors who received each award<br>• Grant numbers awarded to each author<br>• The full name of each funder<br>• URL of each funder website<br>• Did the sponsors or funders play any role in the study design, data collection and analysis, decision to publish, or preparation of the manuscript?<br><br>Did you receive funding for this work?	
**Competing Interests**<br><br>Use the instructions below to enter a competing interest statement for this submission. On behalf of all authors, disclose any competing interests that could be perceived to bias this work—acknowledging all financial support and any other relevant financial or non-financial competing interests.<br><br>This statement is required for submission and **will appear in the published article** if the submission is accepted. Please make sure it is accurate and that any funding sources listed in your Funding Information later in the submission form are also declared in your Financial Disclosure statement.<br><br>View published research articles from *PLOS ONE* for specific examples.	The authors have declared that no competing interests exis

**NO authors have competing interests**<br><br>Enter: *The authors have declared that no competing interests exist.*<br><br>**Authors with competing interests**<br><br>Enter competing interest details beginning with this statement:<br><br>*I have read the journal's policy and the authors of this manuscript have the following competing interests: [insert competing interests here]*<br><br>* typeset	
**Ethics Statement**<br><br>Enter an ethics statement for this submission. This statement is required if the study involved:<br><br>• Human participants<br>• Human specimens or tissue<br>• Vertebrate animals or cephalopods<br>• Vertebrate embryos or tissues<br>• Field research<br><br>Write "N/A" if the submission does not require an ethics statement.<br><br>General guidance is provided below. Consult the submission guidelines for detailed instructions. **Make sure that all information entered here is included in the Methods section of the manuscript.**	Field Research

Powered by Editorial Manager® and ProduXion Manager® from Aries Systems Corporation

**Format for specific study types**<br><br>**Human Subject Research (involving human participants and/or tissue)**<br>• Give the name of the institutional review board or ethics committee that approved the study<br>• Include the approval number and/or a statement indicating approval of this research<br>• Indicate the form of consent obtained (written/oral) or the reason that consent was not obtained (e.g. the data were analyzed anonymously)<br><br>**Animal Research (involving vertebrate animals, embryos or tissues)**<br>• Provide the name of the Institutional Animal Care and Use Committee (IACUC) or other relevant ethics board that reviewed the study protocol, and indicate whether they approved this research or granted a formal waiver of ethical approval<br>• Include an approval number if one was obtained<br>• If the study involved *non-human primates*, add *additional details* about animal welfare and steps taken to ameliorate suffering<br>• If anesthesia, euthanasia, or any kind of animal sacrifice is part of the study, include briefly which substances and/or methods were applied<br><br>**Field Research**<br><br>Include the following details if this study involves the collection of plant, animal, or other materials from a natural setting:<br>• Field permit number<br>• Name of the institution or relevant body that granted permission	
**Data Availability**<br><br>Authors are required to make all data underlying the findings described fully available, without restriction, and from the time of publication. PLOS allows rare exceptions to address legal and ethical concerns. See the PLOS Data Policy and FAQ for detailed information.	Yes - all data are fully available without restriction

A Data Availability Statement describing where the data can be found is required at submission. Your answers to this question constitute the Data Availability Statement and **will be published in the article**, if accepted.<br><br>**Important:** Stating 'data available on request from the author' is not sufficient. If your data are only available upon request, select 'No' for the first question and explain your exceptional situation in the text box.<br><br>Do the authors confirm that all data underlying the findings described in their manuscript are fully available without restriction?	
**Describe where the data may be found in full sentences. If you are copying our sample text, replace any instances of XXX with the appropriate details.**<br><br>• If the data are **held or will be held in a public repository**, include URLs, accession numbers or DOIs. If this information will only be available after acceptance, indicate this by ticking the box below. For example: *All XXX files are available from the XXX database (accession number(s) XXX, XXX.*).<br>• If the data are all contained **within the manuscript and/or Supporting Information files**, enter the following: *All relevant data are within the manuscript and its Supporting Information files.*<br>• If neither of these applies but you are able to provide **details of access elsewhere**, with or without limitations, please do so. For example:<br><br>*Data cannot be shared publicly because of [XXX]. Data are available from the XXX Institutional Data Access / Ethics Committee (contact via XXX) for researchers who meet the criteria for access to confidential data.*<br><br>*The data underlying the results presented in the study are available from (include the name of the third party*	The data are all contained within the manuscript

and contact information or URL).<br>• This text is appropriate if the data are owned by a third party and authors do not have permission to share the data.<br><br>* typeset	
Additional data availability information:	Tick here if the URLs/accession numbers/DOIs will be available only after acceptance of the manuscript for publication so that we can ensure their inclusion before publication.; Tick here if your circumstances are not covered by the questions above and you need the journal's help to make your data available.

Click here to access/download;Manuscript;Manuscript.docx

Nonlinear dynamic wave properties of travelling wave solutions in ion-acoustic waves

S M Yiasir Arafat [a,*], M Asif [a], M M Rahman [a]

[a] Department of Mathematics, Bangladesh University of Engineering and Technology, Dhaka 1000, Bangladesh

E-mail: yiasirarafat28@gmail.com

Abstract

The $(3+1)$-dimensional $mKdV-ZK$ model is an important framework for studying the dynamic behavior of waves in mathematical physics. The goal of this study is to look into more generic travelling wave solutions (TWSs) for the generalized ion-acoustic scenario in three dimensions. Rational, trigonometric, hyperbolic, and exponential solutions are concurrently generated by the new auxiliary equation and the unified techniques applied in the $(3+1)$-dimensional $mKdV-ZK$ model. We created numerous soliton solutions, including kink-shaped soliton solutions, anti-kink-shaped solutions, bell-shaped soliton solutions, periodic solutions, and solitary soliton solutions, for various values of the free parameters in the produced solutions. The attained solutions are displayed geometrically in the surface plot (3-D), contour, and combined two-dimensional (2-D) figures. The combined 2-D figure would make it easier to understand the impact of a wave of speed. These graphical representations are based on specific parameter settings, which help to grasp the model's intricate general behavior. However, the results of this research are compared with the outcomes attainable in published literature executed by other scholars. The results indicate the approach's effectiveness and reliability, making it suitable for widespread use in a range of sophisticated nonlinear models. These techniques successfully generate inventive soliton solutions for various nonlinear models, which are crucial in mathematical physics.

Keywords: $(3+1)$-dimensional $mKdV-ZK$ model, the new auxiliary equation method, the unified method, TWSs, soliton construction.

Introduction

In a wide range of scientific, technical, and technological domains, theoretical answers are superior to experimental results in comprehending physical processes. These real characteristic phenomena of our world are described with the help of nonlinear evolution models (NLEMs). There are a lot of applications of NLEMs in several domains of physics, mathematics, and engineering. A huge number of tasks for NLEMs include plasma physics [1-2], fluid dynamics [3-4], biomathematics [5], nonlinear optics [6-7], shallow water waves [8-9], and many others [10-15]. It is a very challenging task to find the exact solution for NLEMs, but researchers are very interested in discovering potent and effective techniques. Because of the widespread applications and importance of nonlinear models, many scholars have developed a variety of useful tools and approaches to analysis such as, $\left(\frac{G'}{G}\right)$-expansion approach [16], F-expansion method [17], tanh-coth method [18], first integral technique [19], direct algebraic method [20], inverse scattering method [21], sin-Gordon expansion [22], new auxiliary equation approach [23], modified simple equation method [24], Hirota bilinear method [25-26], unified method [27] and so on.

The $(3+1)$-dimensional $mKdV-ZK$ model simplifies the weakly nonlinear ion-acoustic waves in a magnetized electron-positron plasma with equally hot and cool components [28]. In addition, it describes the

evolution of ion-acoustic disturbances in a magnetized plasma with two negative ion components of different temperatures. Therefore, the (3+1)-dimensional $mKdV - ZK$ model provides a base for theoretical research and application across a wide range of scientific areas. The $(3+1)$-dimensional $mKdV - ZK$ [29] model is the following form:

$$u_t + \beta u^2 u_x + u_{xxx} + u_{xyy} + u_{xzz} = 0 \tag{1.1}$$

Where β is an arbitrary constant that measures the nonlinearity's strength, u is a wave function with $x, y, z,$ and t are the independent variables, the subscripts stipulate the partial derivatives. Also, $u^2 u_x$ is a nonlinear term known as cubic nonlinear that indicates the wave profile effect on linear wave propagation and represents cubic relationships.

Throughout history, numerous efficient and compatible methodologies have been used to analyze the $mKdV - ZK$ model such as, the improved fractional sub-equation method [30], the modified Riemann–Liouville derivative, the exp-function, the $\left(\frac{G'}{G}\right)$ -expansion, and the generalized Kudryashov method [31], the improved generalized Riccati equation mapping method [32], the improved Bernoulli sub-equation method [33], the Sardar-sub equation method [34]. the variable separated ODE method [35] and more.

Several researchers have investigated the exact TWSs of numerous NLEMs in various derivative senses by applying the new auxiliary equation and unified methods. To be our best knowledge, it is clear that, researcher has not been found TWSs to the (3+1)-dimensional $mKdV - ZK$ model through new auxiliary equation and unified methods.

Therefore, in the above-mentioned academic study , the core determination of our article is to provide standard, compatible, and stable TWSs to the specified model using these methods. Moreover, we represent the physical illustration as well as the 3-dimensional, contour and combined 2- dimensional graphical depiction are displayed to the investigated solutions. Understanding the (3+1)-dimensional $mKdV - ZK$ model is not only theoretical knowledge; its understanding of wave behavior also enables scientists and engineers to create more efficient communication network and gain a better understanding of wave interactions in natural environments. The outcomes of this study will be significant in clarifying the meaning of the difficult physical phenomena in fluid mechanics, high-frequency plasma physics, marine engineering, ocean physics, and solitary wave theory.

The rest of the manuscript is decorated: In part 2, we discuss the methodology including new auxiliary equation unified methods as well as discuss the application of the stated model via abovementioned methods in part 3. The outcomes of these model are graphically present and give physical explanation with real life significant in part 4. Finally, the paper's conclusion is reached.

2.Overview the Schemes

We will briefly describe two analytical techniques for exploring some soliton solutions of the NLEMs in this section. Now, we utilize the two analytical technique to obtain numerous soliton solutions for the NLEMs. We suppose the NLEMs with variables x, y, z, and t of a function F in the desired construction:

$$F(u, u_x, u_z, u_y, u_t, u_{xx}, u_{xy}, u_{xz}, u_{xt}, u_{yx}, u_{yy}, u_{yz}, u_{yt}, u_{zz}, u_{tt}, \ \dots) = 0 \tag{2.1}$$

In this case, F is a nonlinear polynomial function covering wave function $u(x, y, z, t)$, including its various partial derivatives respect to x, y, z, and t. By using a proper wave transform, we get

$$u(x, y, z, t) = u(\xi), \quad \xi = px + qy + rz - \omega t \tag{2.2}$$

Inserting the Eq. (2.2) into Eq. (2.1), the following ODE form is

$$H(u, \ u', u'', u''', \dots) = 0 \tag{2.3}$$

The symbol $(')$ indicates the derivative with respect to ξ.

2.1 The new auxiliary equation scheme

In this sub-section, we describe the total procedure to the new auxiliary equation method [23] utilize the discover TWSs for the NLEMs presented by Eq. (2.1). We assume that the TWSs to the Eq. (2.3) are the following form:

$$u(\xi) = \sum_{j=0}^{S} k_j a^{jg(\xi)} \tag{2.4}$$

Where $k_j (j = 0, 1, 2, \dots, S)$ are constants to be compute, such that $k_j \neq 0$ and $g(\xi)$ satisfies the following equation

$$g'(\xi) = \frac{1}{\ln(a)}\{m a^{-g(\xi)} + n + l a^{g(\xi)}\} \tag{2.5}$$

The general solutions of the Eq. (2.5) are following:

Case-1: when $n^2 - 4ml < 0$ and $l \neq 0$, $\quad a^{g(\xi)} = \frac{-n}{2l} + \frac{\sqrt{4ml - n^2}}{2l} \tan\left(\frac{\sqrt{4ml-n^2}}{2}\xi\right)$

Or $\quad a^{g(\xi)} = \frac{-n}{2l} + \frac{\sqrt{4ml - n^2}}{2l} \cot\left(\frac{\sqrt{4ml-n^2}}{2}\xi\right)$

Case-2: when $n^2 - 4ml > 0$ and $l \neq 0$, $\quad a^{g(\xi)} = \frac{-n}{2l} - \frac{\sqrt{4ml - n^2}}{2l} \tanh\left(\frac{\sqrt{4ml-n^2}}{2}\xi\right)$

Or $\quad a^{g(\xi)} = \frac{-n}{2l} - \frac{\sqrt{4ml - n^2}}{2l} \coth\left(\frac{\sqrt{4ml-n^2}}{2}\xi\right)$

Case-3: when $n^2 + 4m^2 < 0, \ l \neq 0$ and $l = -m$,

$$a^{g(\xi)} = \frac{n}{2m} - \frac{\sqrt{-n^2 - 4m^2}}{2m} \tan\left(\frac{\sqrt{-n^2-4m^2}}{2}\xi\right)$$

Or $\quad a^{g(\xi)} = \frac{n}{2m} + \frac{\sqrt{-n^2 - 4m^2}}{2m} \cot\left(\frac{\sqrt{-n^2-4m^2}}{2}\xi\right)$

Case-4: when $n^2 + 4m^2 > 0, \ l \neq 0$ and $l = -m$,

$$a^{g(\xi)} = \frac{n}{2m} + \frac{\sqrt{n^2 + 4m^2}}{2m} \tanh\left(\frac{\sqrt{n^2+4m^2}}{2}\xi\right)$$

Or $\quad a^{g(\xi)} = \frac{n}{2m} + \frac{\sqrt{n^2 + 4m^2}}{2m} \coth\left(\frac{\sqrt{n^2+4m^2}}{2}\xi\right)$

Case-5: when $n^2 - 4m^2 < 0$ and $l = m$, $\quad a^{g(\xi)} = \frac{-n}{2m} - \frac{\sqrt{-n^2 + 4m^2}}{2m} \tan\left(\frac{\sqrt{-n^2+4m^2}}{2}\xi\right)$

Or $$a^{g(\xi)} = \frac{-n}{2m} - \frac{\sqrt{-n^2+4m^2}}{2m}\cot\left(\frac{\sqrt{-n^2+4m^2}}{2}\xi\right)$$

Case-6: when $n^2 + 4m^2 > 0$ and $l = m$, $$a^{g(\xi)} = \frac{-n}{2m} - \frac{\sqrt{n^2-4m^2}}{2m}\tanh\left(\frac{\sqrt{n^2-4m^2}}{2}\xi\right)$$

Or $$a^{g(\xi)} = \frac{-n}{2m} - \frac{\sqrt{n^2-4m^2}}{2m}\coth\left(\frac{\sqrt{n^2-4m^2}}{2}\xi\right)$$

Case-7: when $n^2 = 4ml$, $$a^{g(\xi)} = -\frac{2+n\xi}{2l\xi}$$

Case-8: when $ml < 0, n = 0$ and $l \neq 0$, $$a^{g(\xi)} = -\sqrt{-\frac{m}{l}}\tanh\left(\sqrt{-ml}\xi\right)$$

Or $$a^{g(\xi)} = -\sqrt{-\frac{m}{l}}\coth\left(\sqrt{-ml}\xi\right)$$

Case-9: when $n = 0$ and $m = -l$, $$a^{g(\xi)} = \frac{1+e^{(-2l\xi)}}{-1+e^{(-2l\xi)}}$$

Case-10: when $m = l = 0$, $$a^{g(\xi)} = \cosh(n\xi) + \sinh(n\xi)$$

Case-11: when $m = n = C$ and $l = 0$, $$a^{g(\xi)} = e^{C\xi} - 1$$

Case-12: when $n = l = \phi$ and $m = 0$, $$a^{g(\xi)} = \frac{e^{\phi\xi}}{1-e^{\phi\xi}}$$

Case-13: when $n = (m + l)$, $$a^{g(\xi)} = \frac{1-me^{(m-l)\xi}}{1-le^{(m-l)\xi}}$$

Cas-14: when $n = -(m + l)$, $$a^{g(\xi)} = \frac{m-e^{(m-l)\xi}}{l-e^{(m-l)\xi}}$$

Case-15: when $m = 0$, $$a^{g(\xi)} = \frac{ne^{n\xi}}{1-le^{n\xi}}$$

Case-16: when $l = n = m \neq 0$, $$a^{g(\xi)} = \frac{1}{2}\left(\sqrt{3}\tan\left(\frac{\sqrt{3}}{2}m\xi\right) - 1\right)$$

Case-17: when $l = n = 0$, $$a^{g(\xi)} = m\xi$$

Case-18: when $m = n = 0$, $$a^{g(\xi)} = \frac{-1}{l\xi}$$

Case-19: when $l = m$ and $n = 0$, $$a^{g(\xi)} = \tan(m\xi)$$

Case-20: when $l = 0$, $$a^{g(\xi)} = e^{n\xi} - \frac{\phi}{\chi}$$

we determine the positive integer S applying the balancing between the highest order derivatives and highest order nonlinear terms in Eq. (2.3). Substituting Eq. (2.4) and Eq. (2.5) into Eq. (2.3), producing an algebraic equation where the left and right sides are determined by the powers of $a^{jg(\xi)}, (j = 0, 1, 2, \dots)$. After resolving these equations, we get a system of algebraic equation and calculate the values of $k_j (j = 0, 1, 2, \dots), p, q, r, \omega$ and other variables. Finally, the constants $k_j (j = 0, 1, 2, \dots), m, n, l$ are gained, that constants and $g(\xi)$ putting into Eq. (2.4), yield many TWSs from the Eq. (2.1).

2.2 The unified scheme

In this sub-section, we describe the total procedure to the unified method [36] utilize the discover TWSs for the NLEMs presented by Eq. (2.1). Assume that the TWSs to the Eq. (2.3) represented in the following form:

$$u(\xi) = \sum_{j=0}^{S} k_j \varphi(\xi)^j + \sum_{j=1}^{S} l_j \varphi(\xi)^{-j} \quad (2.6)$$

Where $k_j (j = 0,1,2, \dots, S)$ and $l_j (j = 1,2, \dots, S)$, k_s and l_s cannot both be zero simultaneously due to constants that will be examined further. $\varphi = \varphi(\xi)$ satisfy the Riccati differential equation.

$$\varphi'(\xi) = \varphi^2(\xi) + \lambda \qquad (2.7)$$

The general solutions of the Eq. (2.7) are following:

Case-01: when $\lambda < 0$,

$$\varphi(\xi) = \begin{cases} \frac{\sqrt{-(\mathcal{B}^2+\mathcal{R}^2)\lambda} - \mathcal{B}\sqrt{-\lambda}\cosh(2\sqrt{-\lambda}(\xi+h))}{\mathcal{B}\sinh\left(2\sqrt{-\lambda}(\xi+h)\right)+\mathcal{R}} \\ \frac{-\sqrt{-(\mathcal{B}^2+\mathcal{R}^2)\lambda} - \mathcal{B}\sqrt{-\lambda}\cosh(2\sqrt{-\lambda}(\xi+h))}{\mathcal{B}\sinh\left(2\sqrt{-\lambda}(\xi+h)\right)+\mathcal{R}} \\ \sqrt{-\lambda} - \frac{2\mathcal{B}\sqrt{-\lambda}}{\mathcal{B}+\cosh\left(2\sqrt{-\lambda}(\xi+h)\right)-\sinh\left(2\sqrt{-\lambda}(\xi+h)\right)} \\ -\sqrt{-\lambda} + \frac{2\mathcal{B}\sqrt{-\lambda}}{\mathcal{B}+\cosh\left(2\sqrt{-\lambda}(\xi+h)\right)+\sinh\left(2\sqrt{-\lambda}(\xi+h)\right)} \end{cases}$$

Case-02: when $\lambda > 0$,

$$\varphi(\xi) = \begin{cases} \frac{\sqrt{(\mathcal{B}^2-\mathcal{R}^2)\lambda} - \mathcal{B}\sqrt{\lambda}\cos(2\sqrt{\lambda}(\xi+h))}{\mathcal{B}\sin\left(2\sqrt{\lambda}(\xi+h)\right)+\mathcal{R}} \\ \frac{-\sqrt{(\mathcal{B}^2-\mathcal{R}^2)\lambda} - \mathcal{B}\sqrt{\lambda}\cos(2\sqrt{\lambda}(\xi+h))}{\mathcal{B}\sin\left(2\sqrt{\lambda}(\xi+h)\right)+\mathcal{R}} \\ i\sqrt{\lambda} - \frac{2i\mathcal{B}\sqrt{\lambda}}{\mathcal{B}+\cos\left(2\sqrt{\lambda}(\xi+h)\right)-i\sin\left(2\sqrt{\lambda}(\xi+h)\right)} \\ -i\sqrt{\lambda} + \frac{2i\mathcal{B}\sqrt{\lambda}}{\mathcal{B}+\cos\left(2\sqrt{\lambda}(\xi+h)\right)+i\sin\left(2\sqrt{\lambda}(\xi+h)\right)} \end{cases}$$

Case-03: when $\lambda = 0$, $\quad \varphi(\xi) = -\frac{1}{\xi+h}$

where $\mathcal{B}$ and $\mathcal{R}$ are two real type arbitrary parameters, and h is also arbitrary constant.

we determine the positive integer S applying the balancing between the highest order derivatives and highest order nonlinear terms in Eq. (2.3). Substituting Eq. (2.6) and Eq. (2.7) into Eq. (2.3), producing the same powers of $a^j, (j = 0, 1, 2, \dots)$, then setting each coefficient of a^j be zero yield a set of algebraic equation in terms of l_j, c_j and λ. Substituting l_j, c_j and λ into (2.6) with the help of (2.7) we obtained the TWSs of Eq. (2.1) that's directly varied on λ.

3. Mathematical formulation of the model:

In this section, we will apply the new auxiliary equation method and the unified method to $mKdV - ZK$ model to explore TWSs and mathematical analysis. Let us consider the travelling wave transformation

$$u(x, y, z, t) = u(\xi), \ \ \xi = px + qy + rz - \omega t \qquad (3.1)$$

Utilizing Eq. (1.1) with the help of Eq. (3.1), then we get the form:

$$-\omega u' + p\beta u^2 u' + p^3 u''' + pq^2 u''' + pr^2 u''' = 0 \qquad (3.2)$$

Integrating and simplifying of Eq. (3.2), the required form:

$$-\omega u + \frac{1}{3} p\beta u^3 + p^3 u'' + pq^2 u'' + pr^2 u'' = 0 \qquad (3.3)$$

By using the balancing procedure in (3.3), we find $S = 1$.

3.1 Solution analysis through the new auxiliary equation method

We attain the balance value of S from (3.3) the general solution of (2.4) takes the following form:

$$u(\xi) = k_0 + k_1 a^{g(\xi)} \qquad (3.4)$$

where k_0, and k_1 are constants and to be evaluated latter. By inserting Eq.(3.4) and Eq.(2.5) into Eq.(3.3), and then setting the coefficients of $a^{g(\xi)}$ to zero, we may build a set of algebraic equations that Maple can solve to reach the following solution sets:

Set-1: $\omega = \frac{(4p^2ml - p^2n^2 + 4q^2ml - q^2n^2 + 4r^2ml - r^2n^2)p}{2}$, $k_0 = \sqrt{-\frac{3p^2+3q^2+3r^2}{2\beta}}\, n$, $k_1 = 2l\sqrt{-\frac{3p^2+3k^2+3r^2}{2\beta}}$

Set-2: $\omega = \frac{(4p^2ml - p^2n^2 + 4q^2ml - q^2n^2 + 4r^2ml - r^2n^2)p}{2}$, $k_0 = -\sqrt{-\frac{3p^2+3q^2+3m^2}{2\beta}}\, n$, $k_1 - 2l\sqrt{-\frac{3p^2+3q^2+3m^2}{2\beta}}$

For the solution **Set-1** the particular outcomes for Eq. (1.1) are with the help of case-1-case-20:

please correct this sentence.

when $n^2 - 4ml < 0$ and $l \neq 0$, $u_1 = \frac{1}{2}\sqrt{-\frac{6(p^2+q^2+r^2)}{\beta}}\sqrt{4ml - n^2}\tan\left(\frac{\sqrt{4ml-n^2}}{2}\xi\right)$

Or $u_2 = -\frac{1}{2}\sqrt{-\frac{6(p^2+q^2+r^2)}{\beta}}\sqrt{4ml - n^2}\cot\left(\frac{\sqrt{4ml-n^2}}{2}\xi\right)$

when $n^2 - 4ml > 0$ and $l \neq 0$, $u_3 = -\frac{1}{2}\sqrt{-\frac{6(p^2+q^2+r^2)}{\beta}}\sqrt{-4ml + n^2}\tanh\left(\frac{\sqrt{-4ml+n^2}}{2}\xi\right)$

Or $u_4 = -\frac{1}{2}\sqrt{-\frac{6(p^2+q^2+r^2)}{\beta}}\sqrt{-4ml + n^2}\coth\left(\frac{\sqrt{-4ml+n^2}}{2}\xi\right)$

when $n^2 + 4m^2 < 0,\ l \neq 0$ and $l = -m$,

$$u_5 = \frac{1}{2}\sqrt{-\frac{6(p^2+q^2+r^2)}{\beta}}\sqrt{-4m^2 - n^2}\tan\left(\frac{\sqrt{-4m^2-n^2}}{2}\xi\right)$$

Or $u_6 = -\sqrt{-\frac{6(p^2+q^2+r^2)}{\beta}}\sqrt{-4m^2 - n^2}\cot\left(\frac{\sqrt{-4m^2-n^2}}{2}\xi\right)$

when $n^2 + 4m^2 > 0, l \neq 0$ and $l = -m$,

$$u_7 = -\frac{1}{2}\sqrt{-\frac{6(p^2+q^2+r^2)}{\beta}}\sqrt{4m^2 + n^2}\tanh\left(\frac{\sqrt{4m^2+n^2}}{2}\xi\right)$$

Or $u_8 = -\frac{1}{2}\sqrt{-\frac{6(p^2+q^2+r^2)}{\beta}}\sqrt{4m^2 + n^2}\coth\left(\frac{\sqrt{4m^2+n^2}}{2}\xi\right)$

when $n^2 - 4m^2 < 0$ and $l = m$, $u_9 = \frac{1}{2}\sqrt{-\frac{6(p^2+q^2+r^2)}{\beta}}\sqrt{4m^2 - n^2}\tan\left(\frac{\sqrt{4m^2-n^2}}{2}\xi\right)$

Or $u_{10} = -\frac{1}{2}\sqrt{-\frac{6(p^2+q^2+r^2)}{\beta}}\sqrt{4m^2 - n^2}\cot\left(\frac{\sqrt{4m^2-n^2}}{2}\xi\right)$

when $n^2 + 4m^2 > 0$ and $l = m$, $u_{11} = -\frac{1}{2}\sqrt{-\frac{6(p^2+q^2+r^2)}{\beta}}\sqrt{-4m^2 + n^2}\tanh\left(\frac{\sqrt{-4m^2+n^2}}{2}\xi\right)$

Or $u_{12} = -\frac{1}{2}\sqrt{-\frac{6(p^2+q^2+r^2)}{\beta}}\sqrt{-4m^2 + n^2}\coth\left(\frac{\sqrt{-4m^2+n^2}}{2}\xi\right)$

when $n^2 = 4ml$, $u_{13} = -\frac{1}{\xi}\sqrt{-\frac{6(p^2+q^2+r^2)}{\beta}}$

when $ml < 0, n = 0$ and $l \neq 0$, $u_{14} = -l\sqrt{-\frac{6(p^2+q^2+r^2)}{\beta}}\sqrt{-\frac{m}{l}}\tanh(\sqrt{-ml}\,\xi)$

Or $u_{15} = -l\sqrt{-\frac{6(p^2+q^2+r^2)}{\beta}}\sqrt{-\frac{m}{l}}\coth(\sqrt{-ml}\xi)$

when $n = 0$ and $m = -l$, $u_{16} = l\sqrt{-\frac{6(p^2+q^2+r^2)}{\beta}}\left(\frac{1+e^{(-2l\xi)}}{-1+e^{(-2l\xi)}}\right)$

when $n = l = \phi$ and $m = 0$, $u_{17} = -\frac{1}{2}\phi\sqrt{-\frac{6(p^2+q^2+r^2)}{\beta}}\left(\frac{1+e^{\phi\xi}}{-1+e^{\phi\xi}}\right)$

\when $n = (m+l)$, $u_{18} = \frac{1}{2}\sqrt{-\frac{6(p^2+q^2+r^2)}{\beta}}(l-m)\left(\frac{le^{(m-l)\xi}+1}{le^{(m-l)\xi}-1}\right)$

when $n = -(m+l)$, $u_{19} = \frac{1}{2}\sqrt{-\frac{6(p^2+q^2+r^2)}{\beta}}(-m+l)\left(\frac{l+e^{(m-l)\xi}}{-l+e^{(m-l)\xi}}\right)$

when $m = 0$, $u_{20} = -\frac{1}{2}n\sqrt{-\frac{6(p^2+q^2+r^2)}{\beta}}\left(\frac{1+le^{n\xi}}{-1+le^{n\xi}}\right)$

when $l = n = m \neq 0$, $u_{21} = \frac{1}{2}m\sqrt{-\frac{6(p^2+q^2+r^2)}{\beta}}\left\{\left(\sqrt{3}\tan\left(\frac{\sqrt{3}}{2}m\xi\right)-1\right)+1\right\}$

when $m = n = 0$, $u_{22} = -\frac{1}{\xi}\sqrt{-\frac{6(p^2+q^2+r^2)}{\beta}}$

when $l = m$ and $n = 0$, $u_{23} = m\sqrt{-\frac{6(p^2+q^2+r^2)}{\beta}}\tan(m\xi)$

For the solution **Set-2** the particular outcomes for Eq. (1.1) are with the help of case-1-case-20:

when $n^2 - 4ml < 0$ and $l \neq 0$, $u_{24} = -\frac{1}{2}\sqrt{-\frac{6(p^2+q^2+r^2)}{\beta}}\sqrt{4ml-n^2}\tan\left(\frac{\sqrt{4ml-n^2}}{2}\xi\right)$

Or $u_{25} = \frac{1}{2}\sqrt{-\frac{6(p^2+q^2+r^2)}{\beta}}\sqrt{4ml-n^2}\cot\left(\frac{\sqrt{4ml-n^2}}{2}\xi\right)$

when $n^2 - 4ml > 0$ and $l \neq 0$, $u_{26} = \frac{1}{2}\sqrt{-\frac{6(p^2+q^2+r^2)}{\beta}}\sqrt{-4ml+n^2}\tanh\left(\frac{\sqrt{-4ml+n^2}}{2}\xi\right)$

Or $u_{27} = \frac{1}{2}\sqrt{-\frac{6(p^2+q^2+r^2)}{\beta}}\sqrt{-4ml+n^2}\coth\left(\frac{\sqrt{-4ml+n^2}}{2}\xi\right)$

when $n^2 + 4m^2 < 0,\ l \neq 0$ and $l = -m$,

$$u_{28} = -\frac{1}{2}\sqrt{-\frac{6(p^2+q^2+r^2)}{\beta}}\sqrt{-4m^2-n^2}\tan\left(\frac{\sqrt{-4m^2-n^2}}{2}\xi\right)$$

Or $u_{29} = \frac{1}{2}\sqrt{-\frac{6(p^2+q^2+r^2)}{\beta}}\sqrt{-4m^2-n^2}\cot\left(\frac{\sqrt{-4m^2-n^2}}{2}\xi\right)$

when $n^2 + 4m^2 > 0,\ l \neq 0$ and $l = -m$,

$$u_{30} = \frac{1}{2}\sqrt{-\frac{6(p^2+q^2+r^2)}{\beta}}\sqrt{4m^2+n^2}\tanh\left(\frac{\sqrt{4m^2+n^2}}{2}\xi\right)$$

Or $u_{31} = \frac{1}{2}\sqrt{-\frac{6(p^2+q^2+r^2)}{\beta}}\sqrt{4m^2+n^2}\coth\left(\frac{\sqrt{4m^2+n^2}}{2}\xi\right)$

when $n^2 - 4m^2 < 0$ and $l = m$, $u_{32} = -\frac{1}{2}\sqrt{-\frac{6(p^2+q^2+r^2)}{\beta}}\sqrt{4m^2-n^2}\tan\left(\frac{\sqrt{4m^2-n^2}}{2}\xi\right)$

Or $u_{33} = \frac{1}{2}\sqrt{-\frac{6(p^2+q^2+r^2)}{\beta}}\sqrt{4m^2-n^2}\cot\left(\frac{\sqrt{4m^2-n^2}}{2}\xi\right)$

when $n^2 + 4m^2 > 0$ and $l = m$, $u_{34} = \frac{1}{2}\sqrt{-\frac{6(p^2+q^2+r^2)}{\beta}}\sqrt{-4m^2+n^2}\tanh\left(\frac{\sqrt{-4m^2+n^2}}{2}\xi\right)$

Or $u_{35} = \frac{1}{2}\sqrt{-\frac{6(p^2+q^2+r^2)}{\beta}}\sqrt{-4m^2+n^2}\coth\left(\frac{\sqrt{-4m^2+n^2}}{2}\xi\right)$

when $n^2 = 4ml$, $u_{36} = \frac{1}{\xi}\sqrt{-\frac{6(p^2+q^2+r^2)}{\beta}}$

when $ml < 0, n = 0$ and $l \neq 0$, $u_{37} = l\sqrt{-\frac{6(p^2+q^2+r^2)}{\beta}}\sqrt{-\frac{m}{l}}\tanh(\sqrt{-ml}\xi)$

Or $u_{38} = l\sqrt{-\frac{6(p^2+q^2+r^2)}{\beta}}\sqrt{-\frac{m}{l}}\coth(\sqrt{-ml}\xi)$

when $n = 0$ and $m = -l$, $u_{39} = -l\sqrt{-\frac{6(p^2+q^2+r^2)}{\beta}}\left(\frac{1+e^{(-2l\xi)}}{-1+e^{(-2l\xi)}}\right)$

when $n = l = \phi$ and $m = 0$, $u_{40} = \frac{1}{2}\phi\sqrt{-\frac{6(p^2+q^2+r^2)}{\beta}}\left(\frac{1+e^{\phi\xi}}{-1+e^{\phi\xi}}\right)$

when $n = (m + l)$, $u_{41} = -\frac{1}{2}\sqrt{-\frac{6(p^2+q^2+r^2)}{\beta}}(l-m)\left(\frac{le^{(m-l)\xi}+1}{le^{(m-l)\xi}-1}\right)$

when $n = -(m + l)$, $u_{42} = \frac{1}{2}\sqrt{-\frac{6(p^2+q^2+r^2)}{\beta}}(-m+l)\left(\frac{l+e^{(m-l)\xi}}{l-e^{(m-l)\xi}}\right)$

when $m = 0$, $u_{43} = \frac{1}{2}n\sqrt{-\frac{6(p^2+q^2+r^2)}{\beta}}\left(\frac{le^{n\xi}+1}{le^{n\xi}-1}\right)$

when $l = n = m \neq 0$, $u_{44} = \frac{1}{2}\sqrt{-\frac{6(p^2+q^2+r^2)}{\beta}}\left\{\left(\sqrt{3}\tan\left(\frac{\sqrt{3}}{2}m\xi\right)-1\right)l+n\right\}$

when $m = n = 0$, $u_{45} = \frac{1}{\xi}\sqrt{-\frac{6(p^2+q^2+r^2)}{\beta}}$

when $l = m$ and $n = 0$, $u_{46} = -m\sqrt{-\frac{6(p^2+q^2+r^2)}{\beta}}\tan(m\xi)$

3.2 Solution analysis through the unified method

Based upon the number of balance principle S the trial solution of Eq.(3.3) become of the following form:

$$u(\xi) = k_0 + k_1\varphi(\xi) + l_1\varphi(\xi)^{-1} \qquad (3.5)$$

Where k_0, k_1 and l_1 are arbitrary constant and k_1, l_1 cannot both be zero simultaneously. Inserting Eq. (3.5) and Eq. (2.7) into Eq.(3.3), and after that, adjusting the $\varphi(\xi)$ factors to zero, we are able to develop following set of collection:

Set-1: $\omega = 2p^3\lambda + 2pq^2\lambda + 2pr^2\lambda,\ k_0 = 0,\ k_1 = \pm M,\ l_1 = 0.$

Set-2: $\omega = -p(6p^2 + 6q^2 + 6r^2)\lambda + 2p^3\lambda + 2pq^2\lambda + 2pr^2\lambda, k_0 = 0, k_1 = M, l_1 = M\lambda.$

Set-3: $\omega = p(6p^2 + 6q^2 + 6r^2)\lambda + 2p^3\lambda + 2pq^2\lambda + 2pr^2\lambda, k_0 = 0, k_1 = M, k_1 = -M\lambda.$

Set-4: $\omega = p(6p^2 + 6q^2 + 6r^2)\lambda + 2p^3\lambda + 2pq^2\lambda + 2pr^2\lambda, k_0 = 0, k_1 = -M, l_1 = M\lambda.$

Set-5: $\omega = -p(6p^2 + 6q^2 + 6r^2)\lambda + 2p^3\lambda + 2pq^2\lambda + 2pr^2\lambda, k_0 = 0, k_1 = -M, l_1 = -M\lambda.$

Set-6: $\omega = 2p^3\lambda + 2pq^2\lambda + 2pr^2\lambda,\ k_0 = 0,\ k_1 = 0,\ l_1 = \pm M\lambda.$

Where $M = \sqrt{-\frac{6p^2+6q^2+6r^2}{\beta}}$

For the solution **Set-1** the particular outcomes for Eq. (1.1) are with the help of case-1-case-3:

when $\lambda < 0$,

$$u_{47} = \frac{\pm M\left(\sqrt{-(\mathcal{B}^2+\mathcal{R}^2)\lambda} - \mathcal{B}\sqrt{-\lambda}\cosh(2\sqrt{-\lambda}(\xi+h))\right)}{\mathcal{B}\sinh\left(2\sqrt{-\lambda}(\xi+h)\right)+\mathcal{R}}$$

$$u_{48} = \frac{\pm M\left(-\sqrt{-(\mathcal{B}^2+\mathcal{R}^2)\lambda} - \mathcal{B}\sqrt{-\lambda}\cosh\left(2\sqrt{-\lambda}(\xi+h)\right)\right)}{\mathcal{B}\sinh\left(2\sqrt{-\lambda}(\xi+h)\right)+\mathcal{R}}$$

$$u_{49} = \pm M\left(\sqrt{-\lambda} - \frac{2\mathcal{B}\sqrt{-\lambda}}{\mathcal{B}+\cosh\left(2\sqrt{-\lambda}(\xi+h)\right)-\sinh\left(2\sqrt{-\lambda}(\xi+h)\right)}\right)$$

$$u_{50} = \pm M\left(-\sqrt{-\lambda} + \frac{2\mathcal{B}\sqrt{-\lambda}}{\mathcal{B}+\cosh\left(2\sqrt{-\lambda}(\xi+h)\right)+\sinh\left(2\sqrt{-\lambda}(\xi+h)\right)}\right)$$

when $\lambda > 0$,

$$u_{51} = \frac{\pm M\left(\sqrt{(\mathcal{B}^2-\mathcal{R}^2)\lambda} - \mathcal{B}\sqrt{\lambda}\cos\left(2\sqrt{\lambda}(\xi+h)\right)\right)}{\mathcal{B}\sin\left(2\sqrt{\lambda}(\xi+h)\right)+\mathcal{R}}$$

$$u_{52} = \frac{\pm M\left(-\sqrt{(\mathcal{B}^2-\mathcal{R}^2)\lambda} - \mathcal{B}\sqrt{\lambda}\cos(2\sqrt{\lambda}(\xi+h))\right)}{\mathcal{B}\sin\left(2\sqrt{\lambda}(\xi+h)\right)+\mathcal{R}}$$

$$u_{53} = \pm M\left(i\sqrt{\lambda} - \frac{2i\mathcal{B}\sqrt{\lambda}}{\mathcal{B}+\cos\left(2\sqrt{\lambda}(\xi+h)\right)-i\sin\left(2\sqrt{\lambda}(\xi+h)\right)}\right)$$

$$u_{54} = \pm M\left(-i\sqrt{\lambda} + \frac{2i\mathcal{B}\sqrt{\lambda}}{\mathcal{B}+\cos\left(2\sqrt{\lambda}(\xi+h)\right)+i\sin\left(2\sqrt{\lambda}(\xi+h)\right)}\right)$$

when $\lambda = 0$,

$$u_{55} = \frac{\pm M}{(\xi+h)}$$

For the solution **Set-2** the particular outcomes for Eq. (1.1) are with the help of case-1-case-3:

when $\lambda < 0$,

$$u_{56} = \frac{M\left(\sqrt{-(\mathcal{B}^2+\mathcal{R}^2)\lambda} - \mathcal{B}\sqrt{-\lambda}\cosh\left(2\sqrt{-\lambda}(\xi+h)\right)\right)}{\mathcal{B}\sinh\left(2\sqrt{-\lambda}(\xi+h)\right)+\mathcal{R}} + \frac{M\lambda\left(\mathcal{B}\sinh\left(2\sqrt{-\lambda}(\xi+h)\right)+\mathcal{R}\right)}{\sqrt{-(\mathcal{B}^2+\mathcal{R}^2)\lambda} - \mathcal{B}\sqrt{-\lambda}\cosh\left(2\sqrt{-\lambda}(\xi+h)\right)}$$

$$u_{57} = \frac{M\left(-\sqrt{-(\mathcal{B}^2+\mathcal{R}^2)\lambda} - \mathcal{B}\sqrt{-\lambda}\cosh\left(2\sqrt{-\lambda}(\xi+h)\right)\right)}{\mathcal{B}\sinh\left(2\sqrt{-\lambda}(\xi+h)\right)+\mathcal{R}} + \frac{M\lambda\left(\mathcal{B}\sinh\left(2\sqrt{-\lambda}(\xi+h)\right)+\mathcal{R}\right)}{-\sqrt{-(\mathcal{B}^2+\mathcal{R}^2)\lambda} - \mathcal{B}\sqrt{-\lambda}\cosh\left(2\sqrt{-\lambda}(\xi+h)\right)}$$

$$u_{58} = M\sqrt{-\lambda} - \frac{2M\mathcal{B}\sqrt{-\lambda}}{\mathcal{B}+\cosh\left(2\sqrt{-\lambda}(\xi+h)\right)-\sinh\left(2\sqrt{-\lambda}(\xi+h)\right)} + \frac{M\lambda}{\sqrt{-\lambda} - \frac{2\mathcal{B}\sqrt{-\lambda}}{\mathcal{B}+\cosh\left(2\sqrt{-\lambda}(\xi+h)\right)-\sinh\left(2\sqrt{-\lambda}(\xi+h)\right)}}$$

$$u_{59} = -M\sqrt{-\lambda} + \frac{2M\mathcal{B}\sqrt{-\lambda}}{\mathcal{B}+\cosh\left(2\sqrt{-\lambda}(\xi+h)\right)+\sinh\left(2\sqrt{-\lambda}(\xi+h)\right)} + \frac{M\lambda}{-\sqrt{-\lambda} + \frac{2\mathcal{B}\sqrt{-\lambda}}{\mathcal{B}+\cosh\left(2\sqrt{-\lambda}(\xi+h)\right)+\sinh\left(2\sqrt{-\lambda}(\xi+h)\right)}}$$

when $\lambda > 0$,

$$u_{60} = \frac{M\left(\sqrt{((\mathcal{B}^2-\mathcal{R}^2)\lambda} - \mathcal{B}\sqrt{\lambda}\cos\left(2\sqrt{\lambda}(\xi+h)\right)\right)}{\mathcal{B}\sin\left(2\sqrt{\lambda}(\xi+h)\right)+\mathcal{R}} + \frac{M\lambda\left(\mathcal{B}\sin\left(2\sqrt{\lambda}(\xi+h)\right)+\mathcal{R}\right)}{\sqrt{((\mathcal{B}^2-\mathcal{R}^2)\lambda} - \mathcal{B}\sqrt{\lambda}\cos\left(2\sqrt{\lambda}(\xi+h)\right)}$$

$$u_{61} = \frac{M\left(-\sqrt{((\mathcal{B}^2-\mathcal{R}^2)\lambda} - \mathcal{B}\sqrt{\lambda}\cos\left(2\sqrt{\lambda}(\xi+h)\right)\right)}{\mathcal{B}\sin\left(2\sqrt{\lambda}(\xi+h)\right)+\mathcal{R}} + \frac{M\lambda\left(\mathcal{B}\sin\left(2\sqrt{\lambda}(\xi+h)\right)+\mathcal{R}\right)}{-\sqrt{((\mathcal{B}^2-\mathcal{R}^2)\lambda} - \mathcal{B}\sqrt{\lambda}\cos\left(2\sqrt{\lambda}(\xi+h)\right)}$$

$$u_{62}=M\left(i\sqrt{\lambda}-\frac{2i\mathcal{B}\sqrt{\lambda}}{\mathcal{B}+\cos\left(2\sqrt{\lambda}(\xi+h)\right)-i\sin\left(2\sqrt{\lambda}(\xi+h)\right)}\right)+\frac{M\lambda}{\left(i\sqrt{\lambda}-\frac{2i\mathcal{B}\sqrt{\lambda}}{\mathcal{B}+\cos\left(2\sqrt{\lambda}(\xi+h)\right)-i\sin\left(2\sqrt{\lambda}(\xi+h)\right)}\right)}$$

$$u_{63}=M\left(-i\sqrt{\lambda}+\frac{2i\mathcal{B}\sqrt{\lambda}}{\mathcal{B}+\cos\left(2\sqrt{\lambda}(\xi+h)\right)+i\sin\left(2\sqrt{\lambda}(\xi+h)\right)}\right)+\frac{M\lambda}{\left(-i\sqrt{\lambda}+\frac{2i\mathcal{B}\sqrt{\lambda}}{\mathcal{B}+\cos\left(2\sqrt{\lambda}(\xi+h)\right)+i\sin\left(2\sqrt{\lambda}(\xi+h)\right)}\right)}$$

when $\lambda=0$,

$$u_{64}=-\frac{M}{\xi+h}-M\lambda(\xi+h)$$

For the solution **Set-3** the particular outcomes for Eq. (1.1) are with the help of case-1-case-3:

when $\lambda<0$,

$$u_{65}=\frac{M\left(\sqrt{-(\mathcal{B}^2+\mathcal{R}^2)\lambda}-\mathcal{B}\sqrt{-\lambda}\cosh\left(2\sqrt{-\lambda}(\xi+h)\right)\right)}{\mathcal{B}\sinh\left(2\sqrt{-\lambda}(\xi+h)\right)+\mathcal{R}}-\frac{M\lambda\left(\mathcal{B}\sinh\left(2\sqrt{-\lambda}(\xi+h)\right)+\mathcal{R}\right)}{\sqrt{-(\mathcal{B}^2+\mathcal{R}^2)\lambda}-\mathcal{B}\sqrt{-\lambda}\cosh\left(2\sqrt{-\lambda}(\xi+h)\right)}$$

$$u_{66}=\frac{M\left(-\sqrt{-(\mathcal{B}^2+\mathcal{R}^2)\lambda}-\mathcal{B}\sqrt{-\lambda}\cosh\left(2\sqrt{-\lambda}(\xi+h)\right)\right)}{\mathcal{B}\sinh\left(2\sqrt{-\lambda}(\xi+h)\right)+\mathcal{R}}-\frac{M\lambda\left(\mathcal{B}\sinh\left(2\sqrt{-\lambda}(\xi+h)\right)+\mathcal{R}\right)}{-\sqrt{-(\mathcal{B}^2+\mathcal{R}^2)\lambda}-\mathcal{B}\sqrt{-\lambda}\cosh\left(2\sqrt{-\lambda}(\xi+h)\right)}$$

$$u_{67}=M\sqrt{-\lambda}-\frac{2M\mathcal{B}\sqrt{-\lambda}}{\mathcal{B}+\cosh\left(2\sqrt{-\lambda}(\xi+h)\right)-\sinh\left(2\sqrt{-\lambda}(\xi+h)\right)}-\frac{M\lambda}{\sqrt{-\lambda}-\frac{2\mathcal{B}\sqrt{-\lambda}}{\mathcal{B}+\cosh\left(2\sqrt{-\lambda}(\xi+h)\right)-\sinh\left(2\sqrt{-\lambda}(\xi+h)\right)}}$$

$$u_{68}=-M\sqrt{-\lambda}+\frac{2M\mathcal{B}\sqrt{-\lambda}}{\mathcal{B}+\cosh\left(2\sqrt{-\lambda}(\xi+h)\right)+\sinh\left(2\sqrt{-\lambda}(\xi+h)\right)}-\frac{M\lambda}{-\sqrt{-\lambda}+\frac{2\mathcal{B}\sqrt{-\lambda}}{\mathcal{B}+\cosh\left(2\sqrt{-\lambda}(\xi+h)\right)+\sinh\left(2\sqrt{-\lambda}(\xi+h)\right)}}$$

when $\lambda>0$,

$$u_{69}=\frac{M\left(\sqrt{((\mathcal{B}^2-\mathcal{R}^2)\lambda}-\mathcal{B}\sqrt{\lambda}\cos\left(2\sqrt{\lambda}(\xi+h)\right)\right)}{\mathcal{B}\sin\left(2\sqrt{\lambda}(\xi+h)\right)+\mathcal{R}}-\frac{M\lambda\left(\mathcal{B}\sin\left(2\sqrt{\lambda}(\xi+h)\right)+\mathcal{R}\right)}{\sqrt{((\mathcal{B}^2-\mathcal{R}^2)\lambda}-\mathcal{B}\sqrt{\lambda}\cos\left(2\sqrt{\lambda}(\xi+h)\right)}$$

$$u_{70}=\frac{M\left(-\sqrt{((\mathcal{B}^2-\mathcal{R}^2)\lambda}-\mathcal{B}\sqrt{\lambda}\cos\left(2\sqrt{\lambda}(\xi+h)\right)\right)}{\mathcal{B}\sin\left(2\sqrt{\lambda}(\xi+h)\right)+\mathcal{R}}-\frac{M\lambda\left(\mathcal{B}\sin\left(2\sqrt{\lambda}(\xi+h)\right)+\mathcal{R}\right)}{-\sqrt{((\mathcal{B}^2-\mathcal{R}^2)\lambda}-\mathcal{B}\sqrt{\lambda}\cos\left(2\sqrt{\lambda}(\xi+h)\right)}$$

$$u_{71}=M\left(i\sqrt{\lambda}-\frac{2i\mathcal{B}\sqrt{\lambda}}{\mathcal{B}+\cos\left(2\sqrt{\lambda}(\xi+h)\right)-i\sin\left(2\sqrt{\lambda}(\xi+h)\right)}\right)-\frac{M\lambda}{\left(i\sqrt{\lambda}-\frac{2i\mathcal{B}\sqrt{\lambda}}{\mathcal{B}+\cos\left(2\sqrt{\lambda}(\xi+h)\right)-i\sin\left(2\sqrt{\lambda}(\xi+h)\right)}\right)}$$

$$u_{72}=M\left(-i\sqrt{\lambda}+\frac{2i\mathcal{B}\sqrt{\lambda}}{\mathcal{B}+\cos\left(2\sqrt{\lambda}(\xi+h)\right)+i\sin\left(2\sqrt{\lambda}(\xi+h)\right)}\right)-\frac{M\lambda}{\left(-i\sqrt{\lambda}+\frac{2i\mathcal{B}\sqrt{\lambda}}{\mathcal{B}+\cos\left(2\sqrt{\lambda}(\xi+h)\right)+i\sin\left(2\sqrt{\lambda}(\xi+h)\right)}\right)}$$

when $\lambda=0$,

$$u_{73}=-\frac{M}{\xi+h}+M\lambda(\xi+h)$$

For the solution **Set-4** the particular outcomes for Eq. (1.1) are with the help of case-1-case-3:

when $\lambda<0$,

$$u_{74}=-\frac{M\left(\sqrt{-(\mathcal{B}^2+\mathcal{R}^2)\lambda}-\mathcal{B}\sqrt{-\lambda}\cosh\left(2\sqrt{-\lambda}(\xi+h)\right)\right)}{\mathcal{B}\sinh\left(2\sqrt{-\lambda}(\xi+h)\right)+\mathcal{R}}+\frac{M\lambda\left(\mathcal{B}\sinh\left(2\sqrt{-\lambda}(\xi+h)\right)+\mathcal{R}\right)}{\sqrt{-(\mathcal{B}^2+\mathcal{R}^2)\lambda}-\mathcal{B}\sqrt{-\lambda}\cosh\left(2\sqrt{-\lambda}(\xi+h)\right)}$$

$$u_{75}=-\frac{M\left(-\sqrt{-(\mathcal{B}^2+\mathcal{R}^2)\lambda}-\mathcal{B}\sqrt{-\lambda}\cosh\left(2\sqrt{-\lambda}(\xi+h)\right)\right)}{\mathcal{B}\sinh\left(2\sqrt{-\lambda}(\xi+h)\right)+\mathcal{R}}+\frac{M\lambda\left(\mathcal{B}\sinh\left(2\sqrt{-\lambda}(\xi+h)\right)+\mathcal{R}\right)}{-\sqrt{-(\mathcal{B}^2+\mathcal{R}^2)\lambda}-\mathcal{B}\sqrt{-\lambda}\cosh\left(2\sqrt{-\lambda}(\xi+h)\right)}$$

$$u_{76}=-M\sqrt{-\lambda}+\frac{2M\mathcal{B}\sqrt{-\lambda}}{\mathcal{B}+\cosh\left(2\sqrt{-\lambda}(\xi+h)\right)-\sinh\left(2\sqrt{-\lambda}(\xi+h)\right)}+\frac{M\lambda}{\sqrt{-\lambda}-\frac{2\mathcal{B}\sqrt{-\lambda}}{\mathcal{B}+\cosh\left(2\sqrt{-\lambda}(\xi+h)\right)-\sinh\left(2\sqrt{-\lambda}(\xi+h)\right)}}$$

$$u_{77}=M\sqrt{-\lambda}-\frac{2M\mathcal{B}\sqrt{-\lambda}}{\mathcal{B}+\cosh\left(2\sqrt{-\lambda}(\xi+h)\right)+\sinh\left(2\sqrt{-\lambda}(\xi+h)\right)}+\frac{M\lambda}{-\sqrt{-\lambda}+\frac{2\mathcal{B}\sqrt{-\lambda}}{\mathcal{B}+\cosh\left(2\sqrt{-\lambda}(\xi+h)\right)+\sinh\left(2\sqrt{-\lambda}(\xi+h)\right)}}$$

when $\lambda>0$,

$$u_{78}=-\frac{M\left(\sqrt{((\mathcal{B}^2-\mathcal{R}^2)\lambda}-\mathcal{B}\sqrt{\lambda}\cos\left(2\sqrt{\lambda}(\xi+h)\right)\right)}{\mathcal{B}\sin\left(2\sqrt{\lambda}(\xi+h)\right)+\mathcal{R}}+\frac{M\lambda\left(\mathcal{B}\sin\left(2\sqrt{\lambda}(\xi+h)\right)+\mathcal{R}\right)}{\sqrt{((\mathcal{B}^2-\mathcal{R}^2)\lambda}-\mathcal{B}\sqrt{\lambda}\cos\left(2\sqrt{\lambda}(\xi+h)\right)}$$

$$u_{79}=-\frac{M\left(-\sqrt{((\mathcal{B}^2-\mathcal{R}^2)\lambda}-\mathcal{B}\sqrt{\lambda}\cos\left(2\sqrt{\lambda}(\xi+h)\right)\right)}{\mathcal{B}\sin\left(2\sqrt{\lambda}(\xi+h)\right)+\mathcal{R}}+\frac{M\lambda\left(\mathcal{B}\sin\left(2\sqrt{\lambda}(\xi+h)\right)+\mathcal{R}\right)}{-\sqrt{((\mathcal{B}^2-\mathcal{R}^2)\lambda}-\mathcal{B}\sqrt{\lambda}\cos\left(2\sqrt{\lambda}(\xi+h)\right)}$$

$$u_{80}=-Mi\sqrt{\lambda}+\frac{2iM\mathcal{B}\sqrt{\lambda}}{\mathcal{B}+\cos\left(2\sqrt{\lambda}(\xi+h)\right)-i\sin\left(2\sqrt{\lambda}(\xi+h)\right)}+\frac{M\lambda}{\left(i\sqrt{\lambda}-\frac{2i\mathcal{B}\sqrt{\lambda}}{\mathcal{B}+\cos\left(2\sqrt{\lambda}(\xi+h)\right)-i\sin\left(2\sqrt{\lambda}(\xi+h)\right)}\right)}$$

$$u_{81}=Mi\sqrt{\lambda}-\frac{2iM\mathcal{B}\sqrt{\lambda}}{\mathcal{B}+\cos\left(2\sqrt{\lambda}(\xi+h)\right)+i\sin\left(2\sqrt{\lambda}(\xi+h)\right)}+\frac{M\lambda}{\left(-i\sqrt{\lambda}+\frac{2i\mathcal{B}\sqrt{\lambda}}{\mathcal{B}+\cos\left(2\sqrt{\lambda}(\xi+h)\right)+i\sin\left(2\sqrt{\lambda}(\xi+h)\right)}\right)}$$

when $\lambda=0$,

$$u_{82}=\frac{M}{\xi+h}-M\lambda(\xi+h)$$

For the solution **Set-5** the particular outcomes for Eq. (1.1) are with the help of case-1-case-3:

when $\lambda<0$,

$$u_{83}=-\frac{M\left(\sqrt{-(\mathcal{B}^2+\mathcal{R}^2)\lambda}-\mathcal{B}\sqrt{-\lambda}\cosh\left(2\sqrt{-\lambda}(\xi+h)\right)\right)}{\mathcal{B}\sinh\left(2\sqrt{-\lambda}(\xi+h)\right)+\mathcal{R}}-\frac{M\lambda\left(\mathcal{B}\sinh\left(2\sqrt{-\lambda}(\xi+h)\right)+\mathcal{R}\right)}{\sqrt{-(\mathcal{B}^2+\mathcal{R}^2)\lambda}-\mathcal{B}\sqrt{-\lambda}\cosh\left(2\sqrt{-\lambda}(\xi+h)\right)}$$

$$u_{84}=-\frac{M\left(-\sqrt{-(\mathcal{B}^2+\mathcal{R}^2)\lambda}-\mathcal{B}\sqrt{-\lambda}\cosh\left(2\sqrt{-\lambda}(\xi+h)\right)\right)}{\mathcal{B}\sinh\left(2\sqrt{-\lambda}(\xi+h)\right)+\mathcal{R}}-\frac{M\lambda\left(\mathcal{B}\sinh\left(2\sqrt{-\lambda}(\xi+h)\right)+\mathcal{R}\right)}{-\sqrt{-(\mathcal{B}^2+\mathcal{R}^2)\lambda}-\mathcal{B}\sqrt{-\lambda}\cosh\left(2\sqrt{-\lambda}(\xi+h)\right)}$$

$$u_{85}=-M\sqrt{-\lambda}+\frac{2M\mathcal{B}\sqrt{-\lambda}}{\mathcal{B}+\cosh\left(2\sqrt{-\lambda}(\xi+h)\right)-\sinh\left(2\sqrt{-\lambda}(\xi+h)\right)}-\frac{M\lambda}{\sqrt{-\lambda}-\frac{2\mathcal{B}\sqrt{-\lambda}}{\mathcal{B}+\cosh\left(2\sqrt{-\lambda}(\xi+h)\right)-\sinh\left(2\sqrt{-\lambda}(\xi+h)\right)}}$$

$$u_{86}=M\sqrt{-\lambda}-\frac{2M\mathcal{B}\sqrt{-\lambda}}{\mathcal{B}+\cosh\left(2\sqrt{-\lambda}(\xi+h)\right)+\sinh\left(2\sqrt{-\lambda}(\xi+h)\right)}-\frac{M\lambda}{-\sqrt{-\lambda}+\frac{2\mathcal{B}\sqrt{-\lambda}}{\mathcal{B}+\cosh\left(2\sqrt{-\lambda}(\xi+h)\right)+\sinh\left(2\sqrt{-\lambda}(\xi+h)\right)}}$$

when $\lambda>0$,

$$u_{87}=-\frac{M\left(\sqrt{((\mathcal{B}^2-\mathcal{R}^2)\lambda}-\mathcal{B}\sqrt{\lambda}\cos\left(2\sqrt{\lambda}(\xi+h)\right)\right)}{\mathcal{B}\sin\left(2\sqrt{\lambda}(\xi+h)\right)+\mathcal{R}}-\frac{M\lambda\left(\mathcal{B}\sin\left(2\sqrt{\lambda}(\xi+h)\right)+\mathcal{R}\right)}{\sqrt{((\mathcal{B}^2-\mathcal{R}^2)\lambda}-\mathcal{B}\sqrt{\lambda}\cos\left(2\sqrt{\lambda}(\xi+h)\right)}$$

$$u_{88}=-\frac{M\left(-\sqrt{((\mathcal{B}^2-\mathcal{R}^2)\lambda}-\mathcal{B}\sqrt{\lambda}\cos\left(2\sqrt{\lambda}(\xi+h)\right)\right)}{\mathcal{B}\sin\left(2\sqrt{\lambda}(\xi+h)\right)+\mathcal{R}}-\frac{M\lambda\left(\mathcal{B}\sin\left(2\sqrt{\lambda}(\xi+h)\right)+\mathcal{R}\right)}{-\sqrt{((\mathcal{B}^2-\mathcal{R}^2)\lambda}-\mathcal{B}\sqrt{\lambda}\cos\left(2\sqrt{\lambda}(\xi+h)\right)}$$

$$u_{89=}-Mi\sqrt{\lambda}+\frac{2iM\mathcal{B}\sqrt{\lambda}}{\mathcal{B}+\cos\left(2\sqrt{\lambda}(\xi+h)\right)-i\sin\left(2\sqrt{\lambda}(\xi+h)\right)}-\frac{M\lambda}{\left(i\sqrt{\lambda}-\frac{2i\mathcal{B}\sqrt{\lambda}}{\mathcal{B}+\cos\left(2\sqrt{\lambda}(\xi+h)\right)-i\sin\left(2\sqrt{\lambda}(\xi+h)\right)}\right)}$$

$$u_{90}=Mi\sqrt{\lambda}-\frac{2iM\mathcal{B}\sqrt{\lambda}}{\mathcal{B}+\cos\left(2\sqrt{\lambda}(\xi+h)\right)+i\sin\left(2\sqrt{\lambda}(\xi+h)\right)}-\frac{M\lambda}{\left(-i\sqrt{\lambda}+\frac{2i\mathcal{B}\sqrt{\lambda}}{\mathcal{B}+\cos\left(2\sqrt{\lambda}(\xi+h)\right)+i\sin\left(2\sqrt{\lambda}(\xi+h)\right)}\right)}$$

when $\lambda=0$,

$$u_{91} = \frac{M}{\xi+h} + M\lambda(\xi + h)$$

For the solution **Set-6** the particular outcomes for Eq. (1.1) are with the help of case-1-case-3:

when $\lambda < 0$,

$$u_{92} = \frac{\pm M\lambda\left(\mathcal{B}\sin\left(2\sqrt{-\lambda}(\xi+h)\right)+\mathcal{R}\right)}{\sqrt{-((\mathcal{B}^2+\mathcal{R}^2)\lambda} - \mathcal{B}\sqrt{-\lambda}\cos\left(2\sqrt{-\lambda}(\xi+h)\right)}$$

$$u_{93} = \frac{\pm M\lambda\left(\mathcal{B}\sin\left(2\sqrt{-\lambda}(\xi+h)\right)+\mathcal{R}\right)}{-\sqrt{-((\mathcal{B}^2+\mathcal{R}^2)\lambda} - \mathcal{B}\sqrt{-\lambda}\cos\left(2\sqrt{-\lambda}(\xi+h)\right)}$$

$$u_{94} = \frac{\pm M\lambda}{\sqrt{-\lambda} - \frac{2\mathcal{B}\sqrt{-\lambda}}{\mathcal{B}+\cosh\left(2\sqrt{-\lambda}(\xi+h)\right)-\sin\left(2\sqrt{-\lambda}(\xi+h)\right)}}$$

$$u_{95} = \frac{\pm M\lambda}{-\sqrt{-\lambda} + \frac{2\mathcal{B}\sqrt{-\lambda}}{\mathcal{B}+\cosh\left(2\sqrt{-\lambda}(\xi+h)\right)+\sin\left(2\sqrt{-\lambda}(\xi+h)\right)}}$$

when $\lambda > 0$,

$$u_{96} = \frac{\pm M\lambda\left(\mathcal{B}\sin\left(2\sqrt{\lambda}(\xi+h)\right)+\mathcal{R}\right)}{\sqrt{((\mathcal{B}^2-\mathcal{R}^2)\lambda} - \mathcal{B}\sqrt{\lambda}\cos\left(2\sqrt{\lambda}(\xi+h)\right)}$$

$$u_{97} = \frac{\pm M\lambda\left(\mathcal{B}\sin\left(2\sqrt{\lambda}(\xi+h)\right)+\mathcal{R}\right)}{-\sqrt{((\mathcal{B}^2-\mathcal{R}^2)\lambda} - \mathcal{B}\sqrt{\lambda}\cos\left(2\sqrt{\lambda}(\xi+h)\right)}$$

$$u_{98} = \frac{\pm M\lambda}{i\sqrt{\lambda} - \frac{2i\mathcal{B}\sqrt{\lambda}}{\mathcal{B}+\cos\left(2\sqrt{\lambda}(\xi+h)\right)-i\sin\left(2\sqrt{\lambda}(\xi+h)\right)}}$$

$$u_{99} = \frac{\pm M\lambda}{i\sqrt{\lambda} - \frac{2i\mathcal{B}\sqrt{\lambda}}{\mathcal{B}+\cos\left(2\sqrt{\lambda}(\xi+h)\right)-i\sin\left(2\sqrt{\lambda}(\xi+h)\right)}}$$

when $\lambda = 0$,

$$u_{100} = \mp M\lambda(\xi + h)$$

4. Result and Discussion

This part is divided into two subparts. Subpart 4.1 shows a graphical illustration of the obtained solutions, and Subpart 4.2 provides a physical description ~~respectively~~. Using MATLAB software, 3D, Contour, and combined 2D wave profiles are made by choosing an appropriate undefined parameter in the (3+1)-dimensional mKdV-ZK model. Functions with two-dimensional input and one-dimensional output can be displayed using three-dimensional graphs. In data analysis, these graphs are frequently used to identify the highest and lowest levels in a multidimensional data collection. This part aims to illustrate the solutions discovered during this research. We study the physical use of the wave.

4.1 Graphically illustration

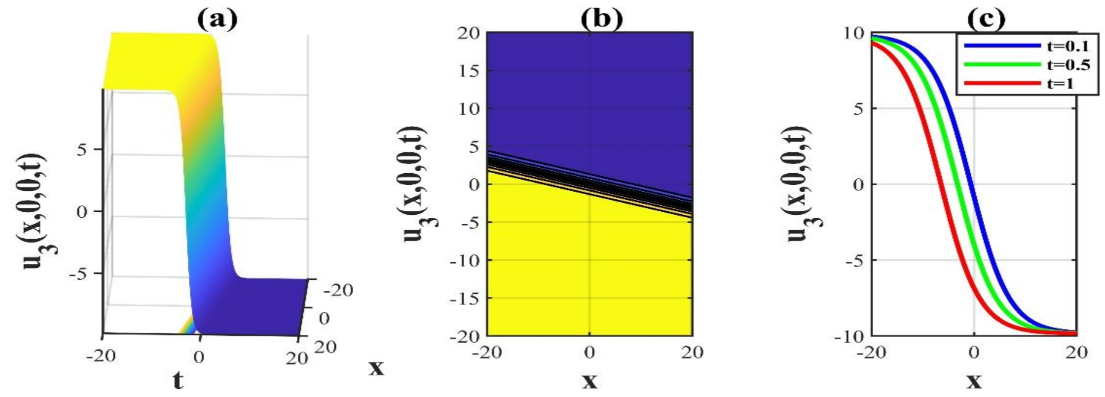

Figs.1 The depictions of the solution $u_3(x, y, z, t)$ for picking unrestricted parameters and we construct a 3D, contour and amalgamated 2D representation over the rang $-20 \leq x, t \leq 20$.

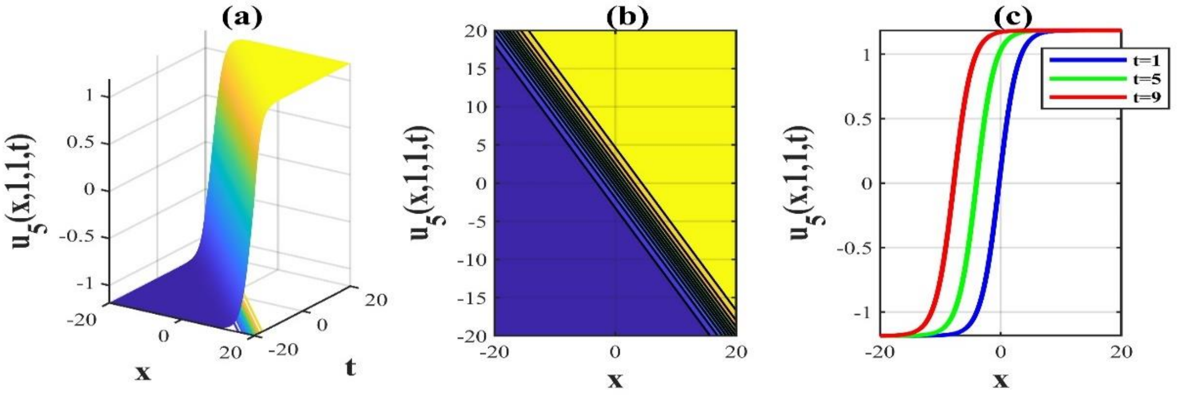

Figs.2 The illustrations of the solution $u_5(x, y, z, t)$ opting for unrestricted constrains and we get a 3D, contour and amalgamated 2D diagram over the rang $-20 \leq x, t \leq 20$.

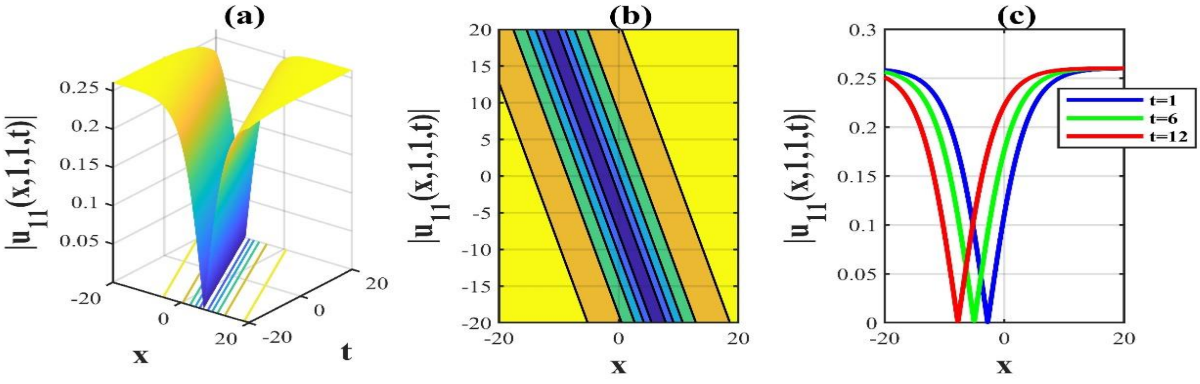

Figs.3 The modulus portrayed of the solution $u_{11}(x, y, z, t)$ for electing free parameters and we attain a 3D, contour and amalgamated 2D diagram over the rang $-20 \leq x, t \leq 20$.

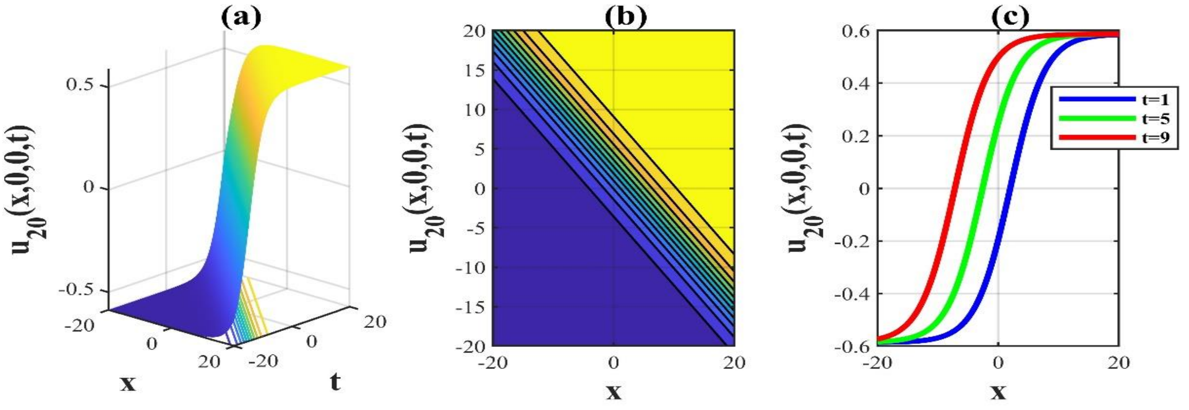

Figs.4 The attributes of the solution $u_{20}(x,y,z,t)$ for picking unrestricted parameters and we build a 3D, contour and combined 2D a schematic over the rang $-20 \le x,t \le 20$.

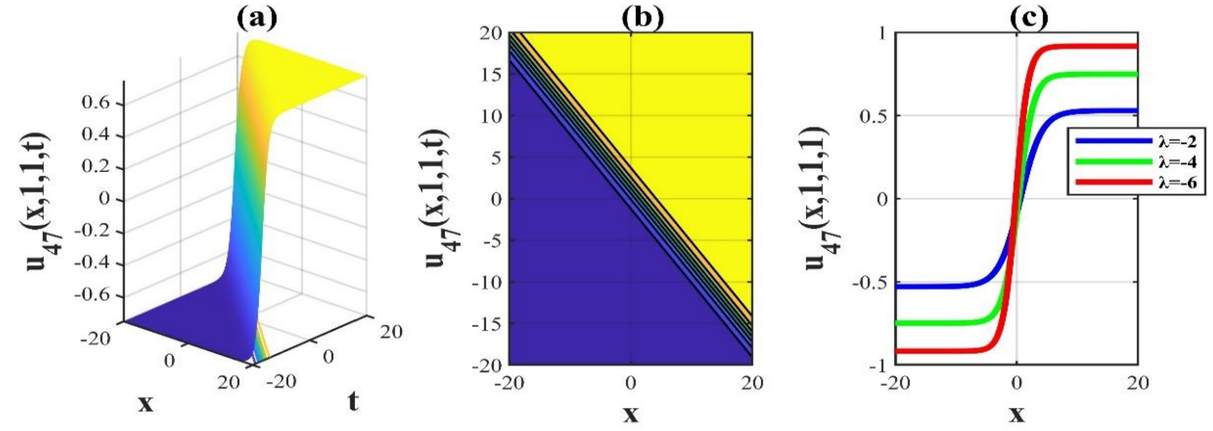

Figs.5 The diagrams of the solution $u_{47}(x,y,z,t)$ for determining parameters and we create a 3D, contour and amalgamated 2D diagram over the rang $-20 \le x,t \le 20$.

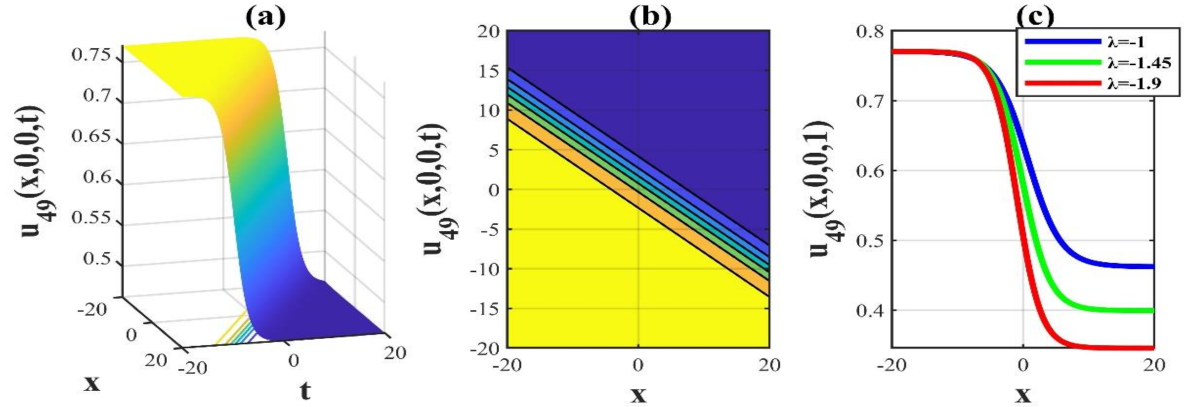

Figs.6 The sketches of the solution $u_{49}(x,y,z,t)$ for picking unrestricted parameters and we construct a 3D, contour and amalgamated 2D representation over the rang $-20 \le x,t \le 20$.

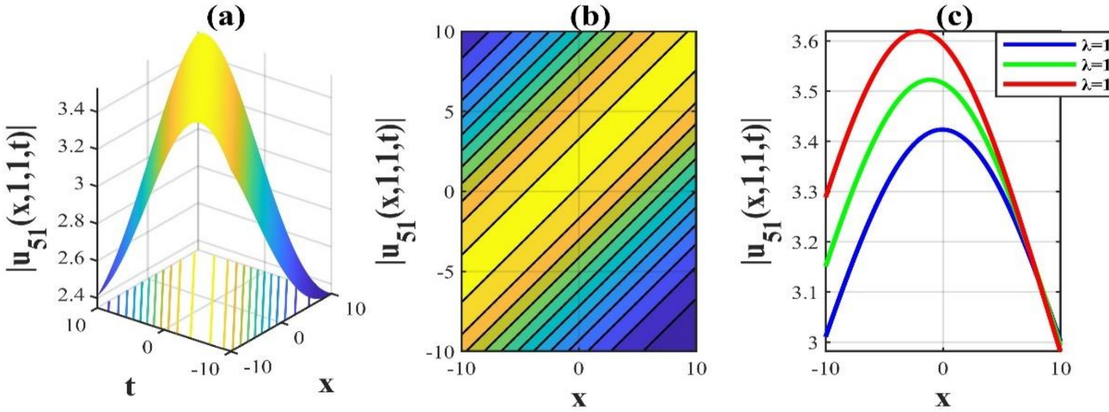

Figs.7 The modulus attributes of the solution $u_{51}(x,y,z,t)$ for choosing parameters and we construct a 3D, contour and combined 2D representation over the rang $-10 \le x,t \le 10$.

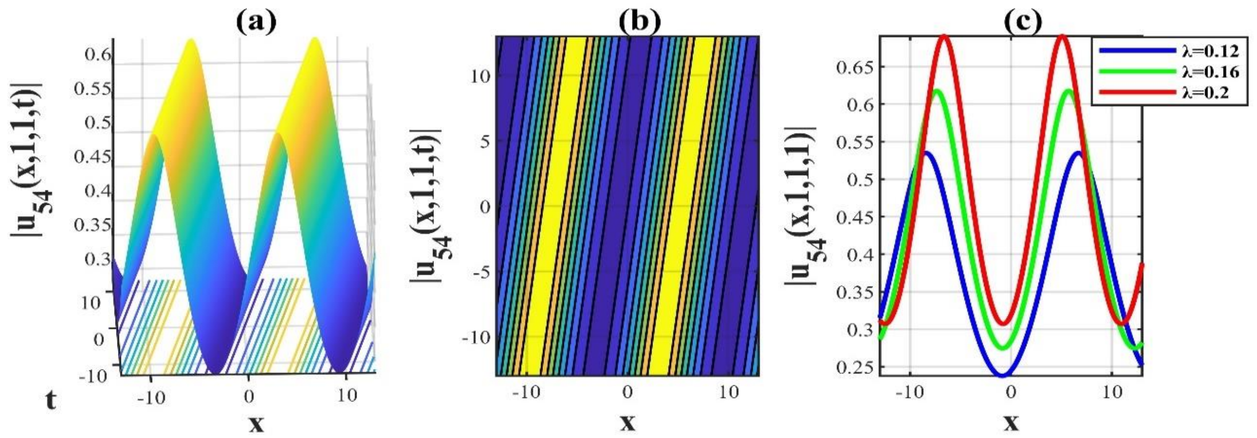

Figs.8 The modulus illustrations of the solution $u_{54}(x,y,z,t)$ for taking free parameters and we attain a 3D, contour and combined 2D diagram over the rang $-10 \leq x,t \leq 10$.

4.2. Physical description

Our main focus was on the wave function $u(x,y,z,t)$ solutions to the (3+1)-dimensional $mKdV - ZK$ models and how the wave profiles are influenced by the parameters. The steady propagation of each wave is depicted in 3D, contour, and combination 2D images. The solution $u_3(x,y,z,t)$ illustrates flat kink shaped soliton for the choosing values of parameters of $p = 0.3, q = 1, r = 1, m = 0.3, n = 2, l = 0.7, \beta = -0.1, y = z = 0,$ is portrayed in Fig. 1(a). and involved contour in Fig. 1(b) are plotted respectively. Fig. 1(c) demonstrates the progression of the waves for different values of $t = 0.1, 0.5, 1$. The desired solution $u_5(\mathrm{x,y,z,t})$ highlights flat anti-kink shaped soliton for taking free parameter of $p = -1, q = 0.3, r = -0.1, m = 0.9, n = 1, l = -0.9, \beta = -1, y = z = 1$ as seen in Fig. 2(a). and equivalent contour in Fig. 2(b) are plotted respectively. Fig. 2(c) indicates the progression of the waves for various values of $t = 0.3, 0.9, 1.5$. The modulus solution $u_{11}(\mathrm{x,y,z,t})$ depicts dark soliton or V-shaped soliton for the suitable values of parameters of $p = 0.3, q = 0.2, r = 0.5, m = 0.25, n = 1.2, l = 0.25, \beta = -10, y = z = 1$ is displayed in Fig. 3(a). and associated contour in Fig. 3(b) are plotted respectively. Fig. 3(c) shows the progression of the waves for different values of $t = 1, 5, 9$. The 2D plot provides a better understanding the effect of free parameters for different values of t and shows various positions of these values. The required solution $u_{20}(\mathrm{x,y,z,t})$ describes smooth anti-kink shaped soliton for the parameters of $p = 0.7, q = -0.3, r = 2, m = -2.5, n = -5.5, l = -3, \beta = -5, y = z = 0$ is portrayed in Fig. 4(a). and involved contour in Fig. 4(b) are plotted respectively. Fig. 4(c) demonstrates the progression of the waves for several values of $t = 1, 5, 9$. The obtain solution $u_{55}(x,y,z,t)$ presents smooth anti-kink soliton for the selecting parametric values of $k = -0.2, l = 0.3, m = 0.1, h = 0, b = 2, r = 1, h = 0, \beta = -6, \lambda = -4, y = z = 1,$ is displayed in Fig. 5(a) and its corresponding contour in fig.5(b) are plotted respectively. Also, Fig. 5(c) shows the progression of the waves for distinguished values of $\lambda = -2, -4, -6$. The desired solution $u_{57}(x,y,z,t)$ shows smooth kink shaped soliton for picking unrestricted parameter of $k = 0.2, l = 0.9, m = 0.2, h = -0.5, b = 0.2, \beta = -9, \lambda = -1, y = z = 0,$ is depicted in Fig. 6(a). and related contour in Fig. 6(b) are plotted respectively. Fig. 6(c) demonstrates the progression of the waves for distinct values of $\lambda = -1, -2, -3$. The modulus solution $u_{59}(\mathrm{x,y,z,t})$ represents bell shaped soliton (which another name bright soliton studying in optical fiber) for the free parameter of $k =$

$0.055,\ l = 0.47,\ m = 0.4, h = 1, b = 1, r = 5,\ \lambda = 1.8, y = z = 1$, is showed in Fig. 7(a). and associated contour in Fig. 7(b) are plotted respectively. Fig. 7(c) shows the progression of the waves for different values of $t = 1.7, 1.8, 1.9$. The absolute solution $u_{62}(x, y, z, t)$ displays M-shaped soliton for the free parameter of $k = 0.6, l = 0.1, m = 0.4, h = 0, b = 5, r = 2, \beta = -3, \lambda = 0.17, y = z = 1$ is displayed in Fig. 8(a). and associated contour in Fig. 8(b) are plotted respectively. Fig. 8(c) shows the progression of the waves for different values of $\lambda = 0.12, 0.16, 0.2$. A stable and durable wave solution is the M-shaped soliton. It can be provide far-reaching data transmission.

Comparison

In this research article, we will discuss comparison between attained solutions and Al-Ghafri et. al [35] solutions. Al-Ghafri et. al [35] studied of the $(3+1)$-dimensional space–time fractional $mKdV - ZK$ equation by the variable separated ODE method. Using the variable separated ODE method, Al-Ghafri et. al [35] have explored Two case included twelve subcase that contains fifty-four solitons' solutions. On the other hand, the new auxiliary equation method used to generate many wave solutions for the $(3+1)$-dimensional $mKdV - ZK$ Model. Both methods have some common solutions shown in Table 1.

Table 1: Comparison between attained solutions with Al-Ghafri et. al [35] solutions.

**Obtained solutions**	**Al-Ghafri et. al [35] solutions**
In Eq.(3.7) taking $p = q = r = l = 1,\ m = 2, n = 3, \beta = -2$, and $u_3(x, y, z, t) = F$, then the solution becomes $F = -\frac{3}{2} tanh\left(\frac{1}{2}\left(x + y + z + \frac{3}{2}t\right)\right)$	In Eq.(44) $u(x, y, z, t) = \frac{\varepsilon}{2}\sqrt{-\frac{6c_2(k_1^2+k_2^2+k_3^2)}{\delta}}\, tanh\left(\frac{\sqrt{c_2}\xi}{2}\right)$ Taking $k_1 = k_2 = k_3 = c_2 = \alpha = 1,\ \varepsilon = -1,\ \delta = -2$ and $u(x, y, z, t) = F$, then the solution becomes $F = -\frac{3}{2} tanh\left(\frac{1}{2}\left(x + y + z + \frac{3}{2}t\right)\right)$
In Eq.(3.8) taking $p = q = r = l = 1, m = 2, n = 3, \beta = -2$, and $u_4(x, y, z, t) = F$ then the solution becomes $F = -\frac{3}{2} coth\left(\frac{1}{2}\left(x + y + z + \frac{3}{2}t\right)\right)$	In Eq.(45) $u(x, y, z, t) = \frac{\varepsilon}{2}\sqrt{-\frac{6c_2(k_1^2+k_2^2+k_3^2)}{\delta}}\, coth\left(\frac{\sqrt{c_2}\xi}{2}\right)$ Taking $k_1 = k_2 = k_3 = c_2 = \alpha = 1,\ \varepsilon = -1,\ \delta = -2$ and $u(x, y, z, t) = F$, then the solution becomes $F = -\frac{3}{2} coth\left(\frac{1}{2}\left(x + y + z + \frac{3}{2}t\right)\right)$
In Eq.(3.12) taking $p = q = r = m = 1, l = -1, n = 2, \beta = -2$ and $u_8(x, y, z, t) = F$, then the solution becomes $F = -3\sqrt{2} coth\left(\sqrt{2}(x + y + z + 12t)\right)$	In Eq(89) $u(x, y, z, t) = 2\varepsilon\sqrt{\frac{3c_2(k_1^2+k_2^2+k_3^2)}{\delta}}\, coth\left(\sqrt{-2c_2}\xi\right)$ Taking $k_1 = k_2 = k_3 = \alpha = 1, c_2 = -1,\ \delta = -18, \varepsilon = -3$ and $u(x, y, z, t) = F$, then the solution becomes $F = -3\sqrt{2} coth\left(\sqrt{2}(x + y + z + 12t)\right)$

In addition to these solutions, we gain forty-three new TWSs $u_1(\xi), u_2(\xi), u_5(\xi) - u_7(\xi)$ and $u_9(\xi) -$ in this article that are not mentioned in Al-Ghafri et. al [35].

In addition, Zafar et. al [37] have also explored twenty solitary wave solutions from the conformable time-fractional $(3+1)$-dimensional $mKdV - ZK$ equation through three integration schemes included as $\exp(\xi)$ function scheme, hyperbolic function scheme, and modified kudryashov scheme. As opposed to generate many wave solutions from the stated equation by the mentioned method in this research. Both approaches share some potential solutions, which are compared and contrasted in Table 2.

Table 2: Comparison between attained solutions with Zafar et. al [37]solutions.

**Obtained solutions**	**Zafar et. al [37] solutions**
In Eq.(3.13) taking $p = q = r = m = l = 1, n = \sqrt{3}, \beta = -2$ and $u_9(x, y, z, t) = F$, then the solution becomes $F = \frac{3}{2} tan\left(\frac{1}{2}\left(x + y + z - \frac{3}{2}t\right)\right)$	In Eq.(3.11) $u_9(\epsilon) = \frac{\sqrt{6}\sqrt{-ek^2 - fp^2 - gq^2}}{2\sqrt{d}}$ $tan\left(\frac{kx + py + qz - l\frac{t^\lambda}{\lambda}}{2}\right)$ Taking $k = p = q = \lambda = e = f = g = 1,\ d = -2$ and $u(x, y, z, t) = F$ then the solution becomes $F = \frac{3}{2} tan\left(\frac{1}{2}\left(x + y + z - \frac{3}{2}t\right)\right)$
In Eq.(3.15) taking $p = q = r = l = m = 1, n = \sqrt{5}, \beta = -2$ and $u_{11}(x, y, z, t) = F$, then the solution becomes $F = -\frac{3}{2} tanh\left(\frac{1}{2}\left(x + y + z + \frac{3}{2}t\right)\right)$	In Eq.(3.7) $u_1(x, y, z, t) = \frac{-\sqrt{6}\sqrt{-ek^2 - fp^2 - gq^2}}{2\sqrt{d}}$ $\tanh\left(\frac{kx + py + qz - \frac{1}{2}(-ek^3 - fkp^2 - gkq^2)\frac{t^\lambda}{\lambda}}{2}\right)$ Taking $k = p = q = \lambda = e = f = g = 1,\ d = -2$ and $u(x, y, z, t) = F$, then the solution becomes $F = -\frac{3}{2} tanh\left(\frac{1}{2}\left(x + y + z + \frac{3}{2}t\right)\right)$

Apart from these solutions, further new forty-four new exact TWSs $u_1(\xi) - u_8(\xi)$, $u_{10}(\xi)$ and $u_{12}(\xi) - u_{46}(\xi)$ are established in this article that are not mentioned in Zafar et. al [37].

5.Conclusion

Through the use of the new auxiliary equation approach and the unified technique, we have achieved the exact and precise travelling wave soliton solutions for the $(3 + 1)$-dimensional $mKdV - Zk$ model. Under certain scenarios, the travelling wave solutions can be expressed as rational, hyperbolic, and trigonometric functions. However, these techniques produce abundantly distinct free parametric values that depict geometrically kink-shaped soliton solutions, anti-kink-shaped solutions, bell-shaped soliton solutions, and periodic solutions. In addition, the reactions of various nonlinearities strengths, wave velocity, and other model factors are investigated. Several of the solutions found are completely fresh and have not been discussed in any of the previous research. To elucidate the attained outcomes, we have looked at surface, contour, and combined 2-D diagrams. For distinct numerical values of parameters, we demonstrated several 2-D graphs, and watching the graph makes it easy to observe the wave velocity. These extensive results can be helpful resources for investigators as they look at the geometrical structure and understand the system's physical interpretation. The outcomes of other research studies that are currently available are compared with the findings of this work. The calculation is straightforward, yields more unique results than other techniques currently used, and has a wider range of applications due to decreased consistency and computational tasks. These techniques yielded precise travelling wave soliton solutions, and they are strongly recommended for future research on nonlinear models, which hold significance in mathematical physics.

6. Reference

[1] Osman MS, and Ghanbari B, “New optical solitary wave solutions of Fokas-Lenells equation in presence of perturbation terms by a novel approach”, Optik, Vol. 175, pp. 328-333, (2018).

[2] Arafat SMY, Islam SMR, Rahman MM, and Saklayen MA, “On nonlinear optical solitons of fractional Biswas-Arshed Model with beta derivative”, Results Phys., Vol. 48, Article Id. 106426, (2023).

[3] Iqbal MA, Akbar MA, and Islam MA, “The nonlinear wave dynamics of fractional foam drainage and Boussinesq equations with Atangana’s beta derivative through analytical solutions”, Results Phys., Vol. 56, Article Id. 107251, (2024).

[4] Adel W, Rezazadeh H, and Inc M, “On numerical solutions of telegraph, viscous, and modified Burgers equations via Bernoulli collocation method”, Sci. Iran., Vol. 31(1), pp. 43-54, (2024).

[5] Shao-Wen Y, Islam ME, Akbar MA, Inc M, Adel M, Osman MS, “Analysis of parametric effects in the wave profile of the variant Boussinesq equation through two analytical approaches”, Open Phys., Vol. 20(1), pp. 778-794, (2022).

[6] Arafat SMY, Khan K, Islam SMR, Rahman MM, “Parametric effects on paraxial nonlinear Schrödinger equation in Kerr media”, Chin. J. Phys, Vol. 83, pp. 361-378, (2023).

[7] Islam SMR, “Bifurcation analysis and exact wave solutions of the nano-ionic currents equation: Via two analytical techniques”, Results Phys., Article Id. 107536, (2024).

[8] Arafat SMY, Rahman MM, Karim MF, Amin MR, “Wave profile analysis of the (2+ 1)-dimensional Konopelchenko–Dubrovsky model in mathematical physics”, Partial Differ. Equ. Appl., Vol. 8, Article Id. 100576, (2023).

[9] Akbar MA, Abdullah FA, Islam MT, Al Sharif MA, Osman MS, “New solutions of the soliton type of shallow water waves and superconductivity models”, Results Phys., Vol. 44, Article Id. 106180, (2023).

[10] Chen J, Pelinovsky DE, and Upsal J, “Modulational instability of periodic standing waves in the derivative NLS equation”, J. Nonlinear Sci., Vol. 31(3), Article Id. 58, (2021).

[11] Osman MS, Rezazadeh H, and Eslami M, “Traveling wave solutions for (3+ 1)-dimensional conformable fractional Zakharov-Kuznetsov equation with power law nonlinearity”, Nonlinear Eng., Vol. 8(1), pp. 559-567, (2019).

[12] Ray AK, and Bhattacharjee JK, “Standing and travelling waves in the shallow-water circular hydraulic jump”, Phys. Lett. A, Vol. 371(3), pp. 241-248, (2007).

[13] Vivas-Cortez M, Arshed S, Sadaf M, Perveen Z, and Akram G, “Numerical simulations of the soliton dynamics for a nonlinear biological model: Modulation instability analysis”, PloS one, Vol. 18(2), Article Id. 0281318, (2023).

[14] Nakkeeran K, “Optical solitons in erbium doped fibers with higher order effects”, Phys. Lett. A, Vol. 275(5-6), pp. 415-418, (2000).

[15] Akbar MA, Abdullah FA, Islam MT, Al Sharif MA, and Osman MS, “New solutions of the soliton type of shallow water waves and superconductivity models”, Results Phys., Vol. 44, Article Id. 106180, (2023).

[16] Biswas A, Mirzazadeh M, and Eslami M, "Dispersive dark optical soliton with Schödinger-Hirota equation by (G′/G)-expansion approach in power law medium", Optik, Vol. 125(16), pp. 4215-4218, (2014).

[17] Yıldırım Y, "Optical solitons with Biswas–Arshed equation by F-expansion method", Optik, Vol. 227, Article Id. 165788, (2021).

[18] Wazwaz AM, "Multiple-soliton solutions for the KP equation by Hirota's bilinear method and by the tanh–coth method", Appl. Math. Comput., Vol. 190(1), pp. 633-640, (2007).

[19] Akinyemi L, Mirzazadeh M, Amin Badri S, and Hosseini K, "Dynamical solitons for the perturbated Biswas–Milovic equation with Kudryashov's law of refractive index using the first integral method", J. Mod. Opt., Vol. 69(3), pp. 172-182, (2022).

[20] Tian Y, and Liu J, "Direct algebraic method for solving fractional Fokas equation", Therm. Sci., Vol. 25(3 Part B), pp. 2235-2244, (2021).

[21] Bao G, Li P, Lin J, and Triki F, "Inverse scattering problems with multi-frequencies", Inverse Probl. Vol. 31(9), Article Id. 093001, (2015).

[22] Raslan KR, Soliman AA, Ali KK, Gaber M, and Almhdy SR, "Numerical Solution for the Sin-Gordon Equation Using the Finite Difference Method and the Non-Stander Finite Difference Method", Appl. Math., Vol. 17(2), pp. 253-260, (2023).

[23] Islam SMR, Arafat SMY, and Wang H, "Abundant closed-form wave solutions to the simplified modified Camassa-Holm equation", J. Ocean Eng. Sci., Vol. 8(3), pp. 238-245, (2023).

[24] Kaplan M, and Bekir A, "The modified simple equation method for solving some fractional-order nonlinear equations", Pramana, Vol. 87, pp. 1-5, (2016).

[25] Wang S, "Novel soliton solutions of CNLSEs with Hirota bilinear method", J Opt., Vol. 1-6, (2023).

[26] Ma WX, "N-soliton solution and the Hirota condition of a (2+ 1)-dimensional combined equation", Math Comput Simul., Vol. 190, pp. 270-279, (2021).

[27] Xiao F, Li G, Jiang D, Xie Y, Yun J, Liu Y, and Fang Z, "An effective and unified method to derive the inverse kinematics formulas of general six-DOF manipulator with simple geometry". Mech Mach Theory., Vol. 159, Article Id. 104265, (2021).

[28] Das KP, and Verheest F, "Ion-acoustic solitons in magnetized multi-component plasmas including negative ions", J. plasma phy., Vol. 41(1), pp. 139-155, (1989).

[29] Nonlaopon K, Mann N, Kumar S, Rezaei S, and Abdou MA, "A variety of closed-form solutions, Painlevé analysis, and solitary wave profiles for modified KdV–Zakharov–Kuznetsov equation in (3+ 1)-dimensions", Results Phys., Vol. 36, Article Id. 105394, (2022).

[30] Sahoo S, and Ray SS, "Improved fractional sub-equation method for (3+ 1)-dimensional generalized fractional KdV–Zakharov–Kuznetsov equations", Comput. Math. with Appl., Vol. 70(2), pp. 158-166, (2015).

[31] Guner O, Aksoy E, Bekir A, and Cevikel AC, "Different methods for (3+ 1)-dimensional space–time fractional modified KdV–Zakharov–Kuznetsov equation", Comput. Math. with Appl., Vol. 71(6), pp. 1259-1269, (2016).

[32] Rehman HU, Awan AU, Hassan, AM, and Razzaq S, "Analytical soliton solutions and wave profiles of the (3+ 1)-dimensional modified Korteweg–de Vries–Zakharov–Kuznetsov equation", Results Phys., Vol. 52, Article Id. 106769, (2023).

[33] Yel G, Sulaiman TA, and Baskonus HM, "On the complex solutions to the (3+ 1)-dimensional conformable fractional modified KdV–Zakharov–Kuznetsov equation", Mod Phys Lett B, Vol. 34(05), Article Id. 2050069, (2020).

[34] Yasin S, Khan A, Ahmad S, and Osman MS, "New exact solutions of (3+ 1)-dimensional modified KdV-Zakharov-Kuznetsov equation by Sardar-subequation method", Opt Quantum Electron, Vol. 56(1), Article Id. 90, (2024).

[35] Al-Ghafri KS, and Rezazadeh H, "Solitons and other solutions of (3+ 1)-dimensional space–time fractional modified KdV–Zakharov–Kuznetsov equation", AMNS, Vol. 4(2), pp. 289-304, (2019).

[36] Akcagil S, and Aydemir T, "A new application of the unified method", New trend math. sci., Vol. 6(1), pp.185-189, (2018).

[37] Zafar A, and Bekir A, "EXPLORING THE CONFORMABLE TIME-FRACTIONAL (3+1)-DIMENSIONAL MODIFIED KORTEWEG-DEVRIES-ZAKHAROV-KUZNETSOV EQUATION VIA THREE INTEGRATION SCHEMES", J. Appl. Anal. Comput., Vol. 11(1), pp. 161-175, (2021).
