# Supplementary material for: Nonlinear dynamic wave properties of travelling wave solutions in in (3+1)-dimensional mKdV−ZK model
Source: PLoS One. 2025 Jan 8;20(1):e0306734. doi: 10.1371/journal.pone.0306734 (PMC11709309; doi:10.1371/journal.pone.0306734)
Supplement: Attachment — Submitted filename: Review Report PONE-D-24-25524.docx [file pone.0306734.s003.docx]

**Report**

I read this paper in detail and the idea of this paper is interesting. However, I suggest the following issues should be revised and modified before it can be considered for publication. My comments are as follows:

1. The abstract should be improved. The author should discuss the significance and advantages of this study according to the title of the manuscript.

2. The physical significance of the model and the phenomenon should be described in the manuscript.

3. A comparative study should be included. What is your innovative work? The authors should add a section and compare your results with others authors results in literature.

4. What are previous studies on the governing model?

5. Please confirm the punctuation mark for each equation, please check the all equations to make sure they are correct and also dot or commas are missing in many equations.

6. The Physical explanation of graphical results are not sufficient. Furthermore, relevant applications should be added to make this article to attract the attention of multidisciplinary audience.

7. What is the reason behind choosing this method though many other methods are also present in the literature? The authors are requested to highlight the same in the section "Introduction".

8. In conclusion section, mention few shortcomings of the proposed method and this should also be expanded to include more comprehensive discussion.

9. For better presentation of the paper, the authors should cite and discuss recent works on the solitons in this field:

- On the Gaussian traveling wave solution to a special kind of Schrödinger equation with logarithmic nonlinearity. Modern Physics Letters B, 36(02), 2150543. doi: 10.1142/S0217984921505436
- Exploration conversations laws, different rational solitons and vibrant type breather wave solutions of the modify unstable nonlinear Schrödinger equation with stability and its multidisciplinary applications, Optical and Quantum Electronics 56 (2024) 420.
- Linear structure and soliton molecules of Sharma-Tasso-Olver-Burgers equation. Physics Letters A, 452, 128430. doi: <https://doi.org/10.1016/j.physleta.2022.128430>
- Exploring fractional-order new coupled Korteweg-de Vries system via improved Adomian decomposition method, Plos one, 19 (5) (2024), e0303426.
- Analytical optical solutions to the nonlinear Zakharov system via logarithmic transformation. Results in Physics, 56, 107298. doi: <https://doi.org/10.1016/j.rinp.2023.107298>

This paper has some technical issues as mentioned above; Therefore, I recommend for publication after major revision in Plos One.
