# Supplementary material for: Nonlinear dynamic wave properties of travelling wave solutions in in (3+1)-dimensional mKdV−ZK model
Source: PLoS One. 2025 Jan 8;20(1):e0306734. doi: 10.1371/journal.pone.0306734 (PMC11709309; doi:10.1371/journal.pone.0306734)
Supplement: Attachment — Submitted filename: Response to Reviewers.docx [file pone.0306734.s004.docx]

# Authors’ Response to the Comments of Reviewer

| **Journal**: | PLOS ONE |
| --- | --- |
| **Manuscript ID** | PONE-S-24-33123 |
| **Title of the Paper**: | Nonlinear dynamic wave properties of travelling wave solutions in ion-acoustic waves |
| **Authors**: | S. M. Yiasir Arafat^[1]*^, M Asif, M. M. Rahman |

Dear Reviewers,

We are really grateful to the editor and all the referees in reviewing this manuscript. We thank to all of them for considering their valuable time and efforts to bring up good points and helpful comments in our work that will greatly improve the manuscript. As indicated below, we have addressed all the general and specific comments provided by the referees and have made necessary changes accordingly to their comments and suggestions. All are highlighted in **yellow color** in the main manuscript. We believed that the revised version can meet the journal publication requirements.

**Response to the comments of reviewer #1**

I have gone through the whole manuscript titled on “Nonlinear dynamic wave properties of travelling wave solutions in ion-acoustic waves”. Overall, the presentation of the article looks very good and but English grammar is not good. The manuscript is potentially publishable, but it requires major revisions, please see the points below to be addressed:

**Comment 1:**

There are many grammatical errors. Kindly check them. (Full stop and commas)

**Response to the comment 1:**

Thank you so much for your comment. We have read the whole paper and founds some grammatical mistakes and corrected them. Also, full stop and commas.

**Comment 2:**

What is the difference between solution sets 1 and 2 on page 12. They only differ in signs.

**Response to the comment 2:**

Thank you very much for your suggestion. Basically, we have addressed the significance variable λ. We attain several types of soliton for the value of positive or negative λ. So, we could not say only differ in sign.

**Comment 3:**

The references are not in a uniform style.

**Response to the comment 3:**

Thank you so much for your comment. We have re-checked all of the references and corrected them according to the journal style as per your suggestion.

**Comment 4:**

What are the applications of the obtained solutions?

**Response to the comment 4:**

Thank you so much for your comment. We have achieved lots of soliton solutions that exist in real world and explain the ion-acoustic scenario as well as the physical phenomena in fluid mechanics, high-frequency plasma physics, marine engineering, ocean physics, and solitary wave theory etc.

**Comment 5:**

What are the advantages and disadvantages of the proposed methods?

**Response to the comment 5:**

The proposed methods can provide many solutions; therefore, the probability of obtaining new soliton solutions is high. In this manuscript, we found many new soliton solutions, which are described in the comparison section. Also, the proposed methods are straightforward, simple, and approachable as well as consist of several free parameters that are helpful to explain the nature of the soliton. On the other hand, certain NLEEs that must have fractional balancing numbers, these methods are not applicable.

**Comment 6:**

The introduction section is lacking drastically with the introduction to the Solitons. The authors are requested to read the following latest articles to enhance the introduction section and cite them properly.

doi.org/10.1155/2014/601961; doi.org/10.3390/sym15020360; doi.org/10.1016/j.asej.2022.101883; doi.org/10.3390/sym14122574; doi.org/10.3390/math10183400.

**Response to the comment 6:**

Thank you so much for your good suggestion. In the revised manuscript, we have discussed your guided literatures in the introduction and added in to the references list which are cited in [29, 41-43].

**Response to the comments of reviewer #2**

PLOS ONE

Manuscript Number: PONE-D-24-25524

This paper discussed the traveling wave solutions of the (3 + 1)-dimensional mKdV-ZK model using the new auxiliary equation method and the unified technique. Some traveling wave solutions in the form of rational, trigonometric, hyperbolic, and exponential functions were obtained. Moreover, some two- and three-dimensional figures were presented for some solutions. However, the authors should consider the following comments :

**Comment 1:**

The title of the paper is more general. I think that it should be changed.

**Response to the comment 1:**

As per your suggestion, we have selected the new title of the paper is “ **Nonlinear dynamic wave properties of travelling wave solutions in** $\left( \boldsymbol{3+1} \right)$**-dimensional** $\boldsymbol{mKdV-ZK}$ **model .**”

**Comment 2:**

In the Abstract, please correct the grammar as I wrote in the attached file.

**Response to the comment 2:**

Thank you very much for your valuable comment. As per your suggestion, we read the entire work corrected the grammar and addressed the innovation [e.g. influence of the nonlinear parameter $\beta$ on wave type, ] in the abstract sections. and highlighted by yellow color.

**Comment 3:**

Many sentences are not consistent with each other and there is no coherence between most sentences. Please check the whole manuscript for this point.

**Response to the comment 3:**

Thank you so much for your comment. We have read the whole paper and found some problems as well as grammatical mistakes and we corrected them according to your comments. We hope that now the manuscript improved the previous one.

**Comment 4:**

The whole manuscript should be checked for typos and grammatical errors. An overall review is needed for fixing the grammatical and typos errors in the manuscript. Please see the attached file.

**Response to the comment 4:**

Thank you so much for your good suggestion. In the revised manuscript, we have corrected some errors and sentences. Now it is suitable for a broad audience.

**Comment 5:**

The obtained results should be double checked.

**Response to the comment 5:**

Thank you very much for your comment. In this paper, we re-checked all of the results and figures, and they are all right.

**Comment 6:**

You need to describe the values of m, n and l in Eq. (2.4). Are they real, complex, etc.?

**Response to the comment 6:**

Thank you so much for your comment. In methodology section, m, n and l are real constant that are mention in revised manuscript.

**Comment 7:**

The determination of the value of S (in Eq. (2.4) ) was described in page 10. Please put it in page 9.

**Response to the comment 7:**

Thank you very much for your comment. According to your comment we have replaced your mentioned sentence in suitable place.

**Comment 8:**

Punctuation marks should be checked throughout the paper, especially after the equations and at the end of the statements. Please see the attached file.

**Response to the comment 8:**

Thank you so much for your comment. We have read the whole paper thoroughly and corrected all Punctuation marks (full stop and commas).

**Comment 9:**

The introduction should be rearranged in a manner to list the various effects considered in the study and their connection to the existing literature. These are some recent studies to include in the introduction covering various effects used in the present manuscript. I suggest some papers such as: <https://doi.org/10.3390/fractalfract7030252>; <https://doi.org/10.1142/S0218348X23401606>; <https://doi.org/10.1080/00036811.2022.2027381>; <https://doi.org/10.32604/cmes.2023.027344>; <https://doi.org/10.1016/j.jksus.2022.102087>; <https://scik.org/index.php/cmbn/article/view/8478>; <https://scik.org/index.php/cmbn/article/view/8477>; <https://doi.org/10.28924/2291-8639-22-2024-51>; <https://doi.org/10.28924/2291-8639-21-2023-100>; <https://doi.org/10.3390/fractalfract7050344>.

**Response to the comment 9:**

Thank you so much for your good suggestion. In the revised manuscript, we have discussed your guided literatures in the introduction and added in to the references list, which are cited between [16-27].

**Comment 10:**

In the comparison’s section, you compared the considered model with a space-time fractional model. Is this selection correct? You need to compare your results with other results obtained on other papers for the same model.

**Response to the comment 10:**

In the “Comparison Section”, we compared our results with two different published articles. we have considered $\left( 3+1 \right)$-dimensional space-time fractional $mKdV-ZK$ model via variable separated ODE method and $\left( 3+1 \right)$-dimensional $mKdV-ZK$ model via exp$\left( \xi\right)$ function scheme. If we consider $\alpha=1$, the $\left( 3+1 \right)$-dimensional space-time fractional $mKdV-ZK$ model will be converted $\left( 3+1 \right)$-dimensional $mKdV-ZK$ model. So, our selection is obviously correct.

**Response to the comments of reviewer #3**

I read this paper in detail and the idea of this paper is interesting. However, I suggest the following issues should be revised and modified before it can be considered for publication. My comments are as follows:

**Comment 1 :**

The abstract should be improved. The author should discuss the significance and advantages of this study according to the title of the manuscript.

**Response to the comment 1 :**

Thank you very much for your valuable comment. As per your suggestion, we read the entire abstract section and addressed the significance and innovation [e.g. influence of the nonlinear parameter β on wave type, ] in the abstract sections.

**Comment 2 :**

The physical significance of the model and the phenomenon should be described in the manuscript.

**Response to the comment 2 :**

Thank you very much for your comment. The $\left( 3+1 \right)$-dimensional $mKdV-ZK$ model describes the magnetized electron-positron plasma, evolution of ion-acoustic disturbances and many others. All of are smoothly discussed in “Introduction Section”.

**Comment 3:**

A comparative study should be included. What is your innovative work? The authors should add a section and compare your results with others authors results in literature.

**Response to the comment 3 :**

In the “Comparison Section”, we compared our results with two different published articles. We have got lots of new solutions that exist in real world. For instant, fluid mechanics, high-frequency plasma physics, marine engineering, ocean physics, and solitary wave theory etc.

**Comment 4:**

What are previous studies on the governing model?

**Response to the comment 4:**

Thank you for your comment. We have read lots of article studies in the governing model. We have discussed [28-35] in the “Introduction Section” and [35,36] in the “Comparison Section”.

**Comment 5:**

Please confirm the punctuation mark for each equation, please check the all equations to make sure they are correct and also dot or commas are missing in many equations.

**Response to the comment 5:**

Thank you so much for your comment. We have read the whole paper, found some grammatical and punctuation mistakes and corrected them. Also, we have re-checked all of the results, they are all right.

**Comment 6:**

The Physical explanation of graphical results are not sufficient. Furthermore, relevant applications should be added to make this article to attract the attention of multidisciplinary audience.

**Response to the comment 6:**

Thank you so much for your good suggestion. In the revised manuscript, we have re-write the Physical explanation of graphical results and application. Now it is suitable for a broad audience.

**Comment 7:**

What is the reason behind choosing this method though many other methods are also present in the literature? The authors are requested to highlight the same in the section "Introduction".

**Response to the comment 7:**

The proposed methods can provide many solutions; therefore, the probability of obtaining new soliton solutions is high. In this manuscript, we found many new soliton solutions, proven by comparison section. Also, the proposed methods are straightforward, simple, and approachable as well as consist of several free parameters that are helpful to explain the nature of the soliton. Also highlighted in “Introduction Section.”

**Comment 8:**

In conclusion section, mention few shortcomings of the proposed method and this should also be expanded to include more comprehensive discussion.

**Response to the comment 8:**

In the revised manuscript, we have discussed your guide and mentioned the shortcomings of the proposed method in the conclusion section. Also, we have extended this section according to your advice.

**Comment 9:**

For better presentation of the paper, the authors should cite and discuss recent works on the solitons in this field:

• On the Gaussian traveling wave solution to a special kind of Schrödinger equation with logarithmic nonlinearity. Modern Physics Letters B, 36(02), 2150543. doi: 10.1142/S0217984921505436

• Exploration conversations laws, different rational solitons and vibrant type breather wave solutions of the modify unstable nonlinear Schrödinger equation with stability and its multidisciplinary applications, Optical and Quantum Electronics 56 (2024) 420.

• Linear structure and soliton molecules of Sharma-Tasso-Olver-Burgers equation. Physics Letters A, 452, 128430. doi: https://doi.org/10.1016/j.physleta.2022.128430

• Exploring fractional-order new coupled Korteweg-de Vries system via improved Adomian decomposition method, Plos one, 19 (5) (2024), e0303426.

• Analytical optical solutions to the nonlinear Zakharov system via logarithmic transformation. Results in Physics, 56, 107298. doi: https://doi.org/10.1016/j.rinp.2023.107298.

**Response to the comment 9:**

Thank you so much for your good suggestion. In the revised manuscript, we have discussed your guided literatures in the introduction and added in to the references list, which are cited between [16-27].

The revised manuscript has been re-submitted to the journal, and we look forward to hearing your positive response.

Thank you very much for your valuable comments and suggestion to improve the manuscript.

With Kindest Regards

S. M. Yiasir Arafat

Corresponding author

E-mail[: yiasirarafat28@gmail.com](mailto::%20yiasirarafat28@gmail.com)

1. * Corresponding author: email[: yiasirarafat28@gmail.com](mailto::%20yiasirarafat28@gmail.com) (Attn: S. M. Yiasir Arafat )
